# Supplementary material for: Creb5 coordinates synovial joint formation with the genesis of articular cartilage
Source: Nat Commun. 2022 Nov 26;13:7295. doi: 10.1038/s41467-022-35010-0 (PMC9701237; doi:10.1038/s41467-022-35010-0)
Supplement: Supplementary file 1 — Supplemental Information [file 41467_2022_35010_MOESM1_ESM.pdf]

Supplementary Materials for:

"Creb5 coordinates synovial joint formation with the genesis of articular cartilage"

Authors: Cheng-Hai Zhang, Yao Gao, Han-Hwa Hung, Zhu Zhuo, Alan J. Grodzinsky and Andrew B. Lassar

List of Supplementary Materials:

Supplementary Figs. 1-11

Supplementary Tables 1-6

Supplementary Figure 1

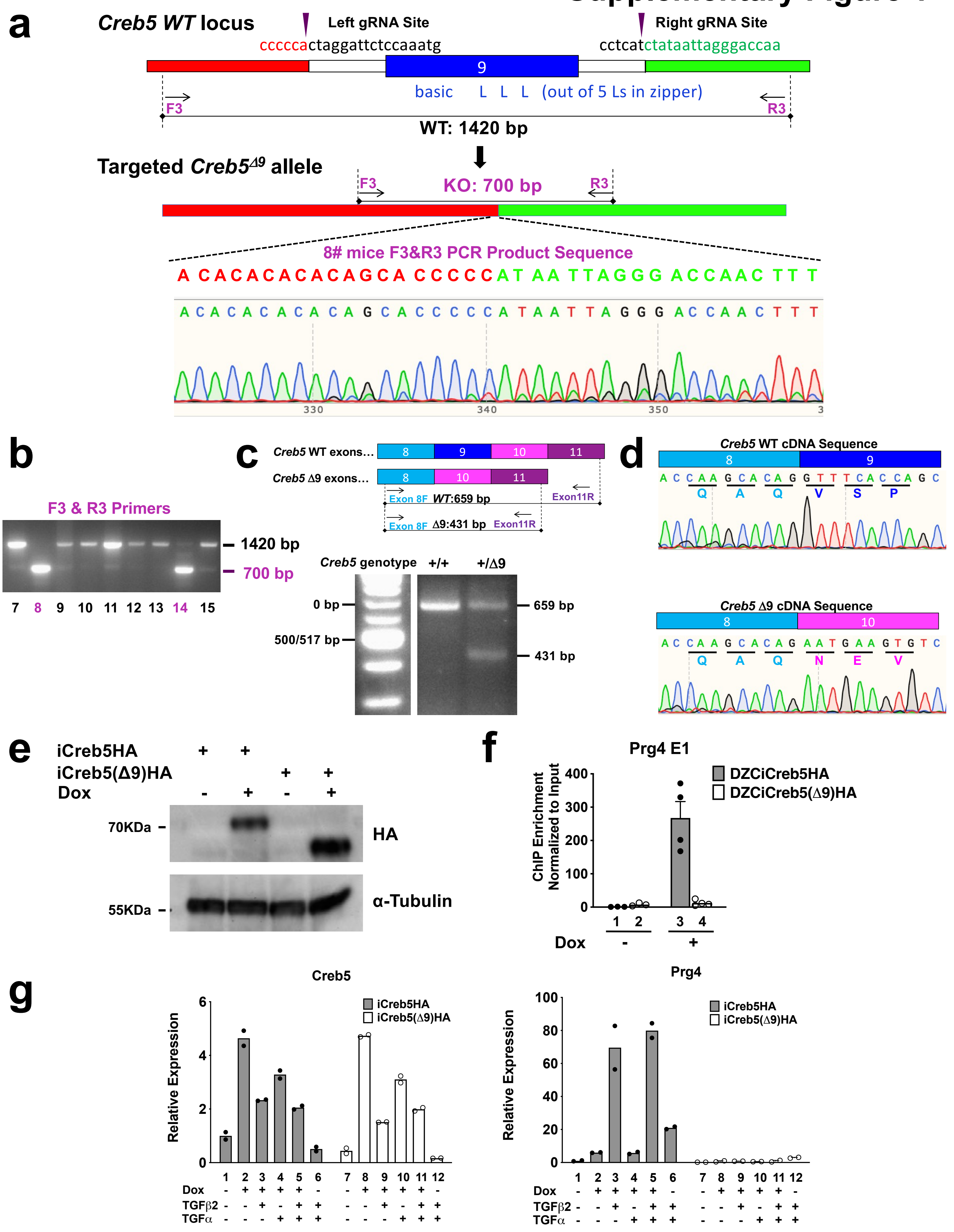

Supplementary Fig 1. Generation of the *Creb5*$^{\Delta 9}$ allele, which lacks an intact DNA binding domain. (a) WT *Creb5* exon9 (blue) and flanking intron sequences depicted with targeting CRISPR guide RNAs. The targeted allele following non-homologous end-joining is depicted and the sequence of the junction in the new chimeric intron that now lies between exon 8 and exon 10 of the *Creb5*$^{\Delta 9}$ allele is displayed (derived from mouse 8). A 700bp PCR amplicon was generated from founder mouse #8 genomic DNA. Sequence analysis of this amplicon derived from genomic DNA revealed that exon 9 was deleted in the *Creb5*$^{\Delta 9}$ allele. (b) Diagnostic PCRs of tail DNA from some of the founder pups born after Cas9 and gRNA injection into one cell embryos is displayed. 33 founder pups derived from these injected embryos were genotyped. Founder mice 8 and 14 contain a *Creb5* allele that has deleted the *Creb5* 9th exon. (c-d) Consistent with loss of exon 9 in the *Creb5*$^{\Delta 9}$ allele, RT-PCR of the Creb5 cDNA (derived from the brain tissue of either adult *WT* or adult *Creb5*$^{\Delta 9/+}$ mice) with primers located in *Creb5* exons 8 and 11 indicated that exon 8 is directly spliced (in frame) to exon 10 in the *Creb5*$^{\Delta 9}$ mRNA. Deletion of exon 9 is predicted to yield an in-frame mutant Creb5 protein, that specifically lacks 76 amino acids that encode a critical part of the bZIP domain of Creb5 and is necessary for direct interaction of Creb5 with DNA targets. (e-f) Bovine deep zone articular chondrocytes (DZCs) were infected with lentivirus encoding either doxycycline-inducible HA-tagged Creb5 (iCreb5-HA) or doxycycline-inducible HA-tagged Creb5(Δ9) (iCreb5(Δ9)-HA). The cells were cultured in medium containing both TGF-α plus TGF-β2 in either the absence or presence of doxycycline, as indicated. (e) Western analysis displays relative expression of iCreb5-HA and iCreb5(Δ9)-HA in the infected DZCs. (f) Chromatin-Immunoprecipitation (ChIP)-PCR evaluating iCreb5-HA or iCreb5(Δ9)-HA occupancy on the E1 enhancer/ATAC-Seq peak [18] in the bovine *Prg4* locus is displayed. A representative ChIP-PCR is displayed. Similar results were obtained in a total of n=3 independent biological repeats. Technical repeats of the PCR analysis of one such experiment are displayed in

the graph. (g) Gene expression was assayed by RT-qPCR in bovine DZCs programmed to express either iCreb5-HA or iCreb5(Δ9)-HA and cultured in doxycycline, TGFα and TGFβ, as indicated. Similar results were obtained in a total of n=3 independent biological repeats. Technical repeats of the PCR analysis are displayed in the graph.

Supplementary Figure 2

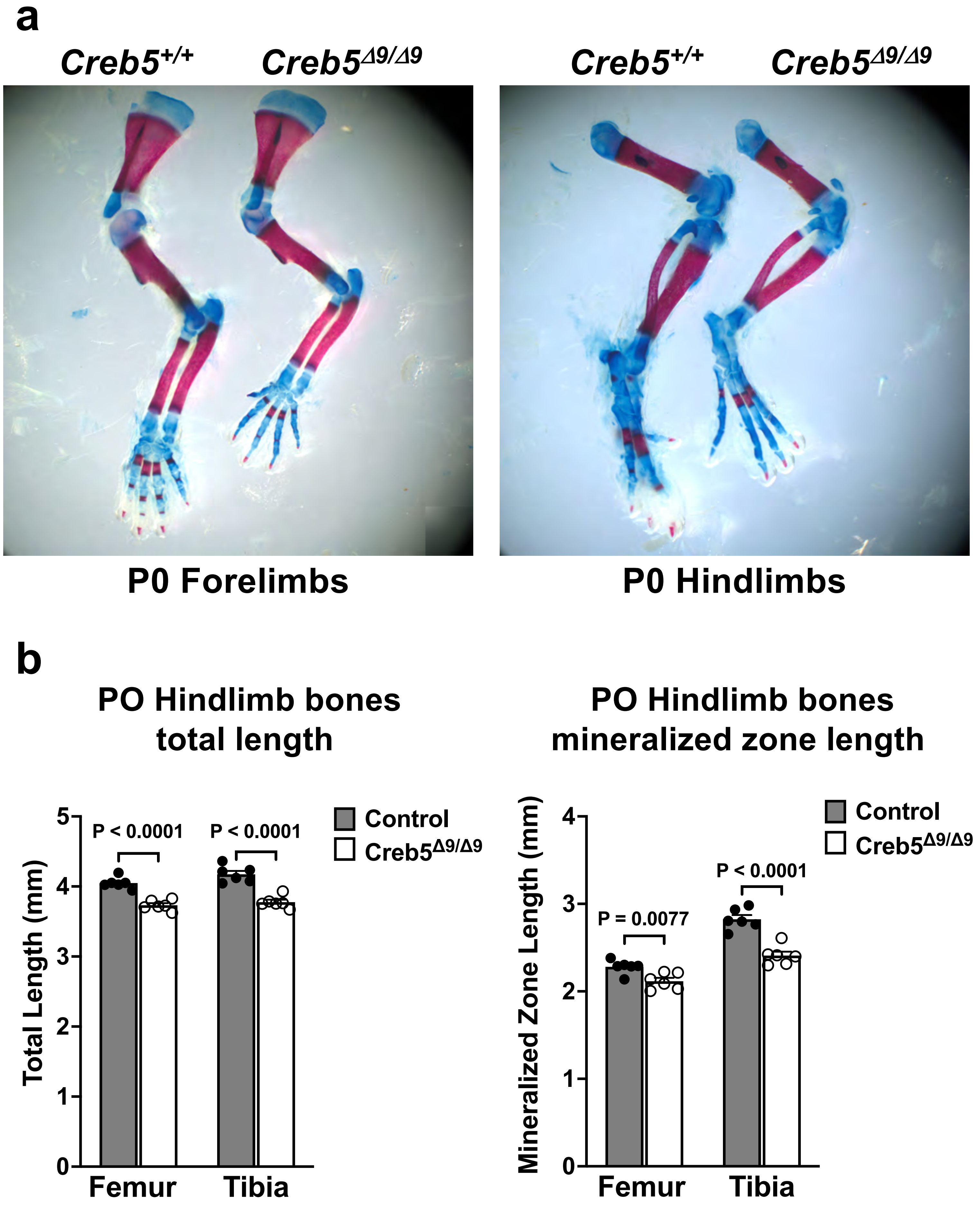

Supplementary Fig 2. Creb5-deficient P0 mice display shorter long bones that WT littermates. (a) Whole mount Alcian Blue/Alizarin Red staining of the appendicular skeleton of P0 *Creb5*$^{\Delta9/\Delta9}$ mice or their WT littermates. (b) Quantitation of either the total length or length of the mineralized region of the femur and tibia of either P0 *Creb5*$^{\Delta9/\Delta9}$ mice or their control littermates (which are either *Creb5*$^{+/\Delta9}$ *or Creb5*$^{+/+}$). Each point represents a hindlimb of a different mouse (n=6). Statistical analysis was a two-sided test without adjustments. P values are indicated and error bar indicates standard error of the mean. Similar results were obtained in n=6 independent biological repeats.

Supplementary Figure 3

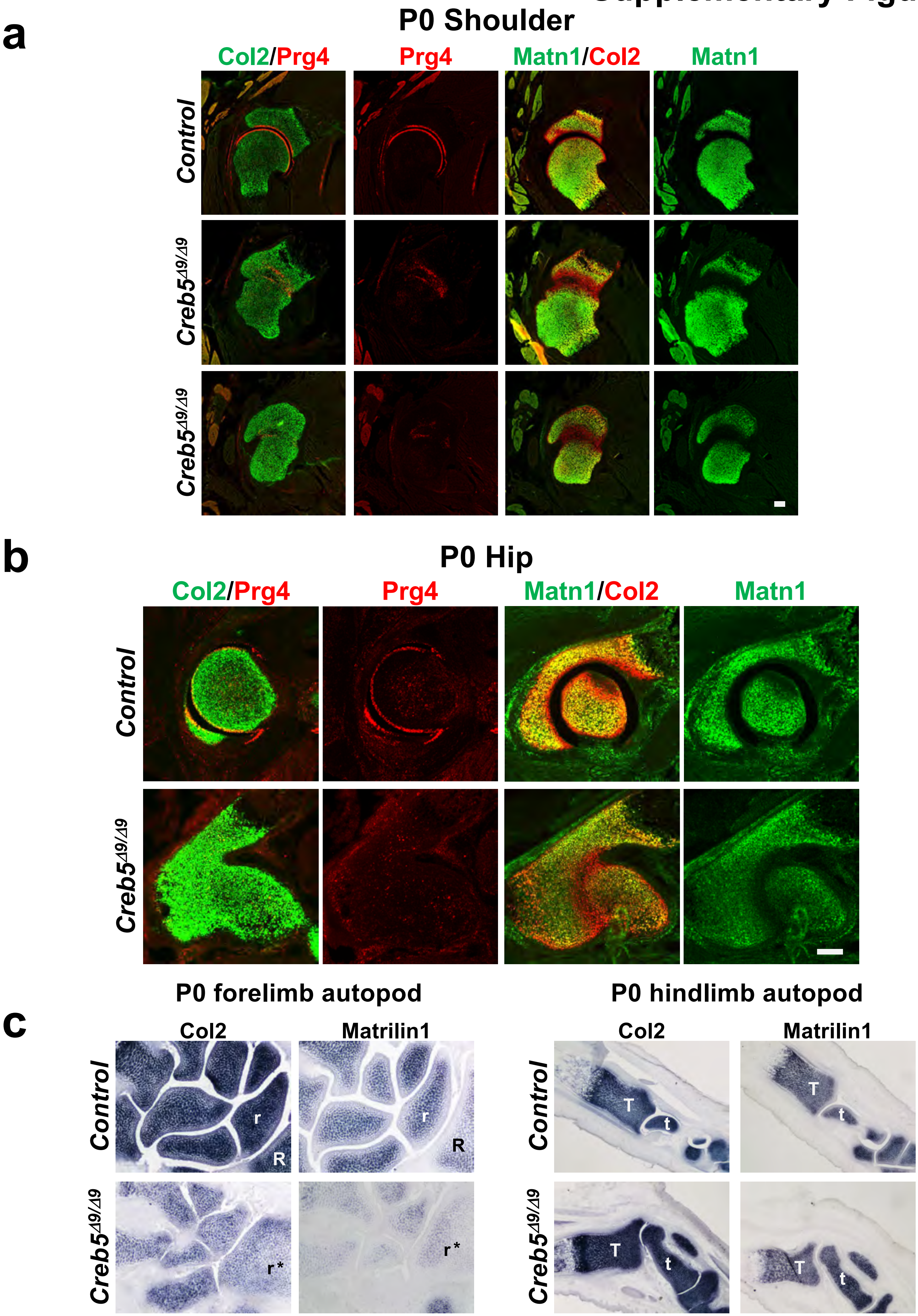

Supplementary Fig 3. Creb5 is necessary for the proper formation of shoulder and hip joints. Gene expression assayed by FISH in serial sections of either the shoulder (a), the hip (b), or the autopods (c) from either a P0 *Creb5*$^{\Delta 9/\Delta 9}$ mouse or a control littermate. While the shoulder, hip, elbow, knee, radiocarpal, ulnocarpal, metacarpophalangeal, metatarsophalangeal, and interphalangeal joints all show defects in their formation, many (but not all) mesopodial joints form in P0 *Creb5*$^{\Delta 9/\Delta 9}$ mice but are not as fully separated from one another as in control littermates. Radius (R), radial element (r), radial element fusion (r*) to both other carpal elements and radius, tibia (T) and talus (t) are indicated. Similar results were obtained in 3 (a, c) or 1 (b) independent biological repeats. Scale bar equals 200 microns.

Supplementary Figure 4

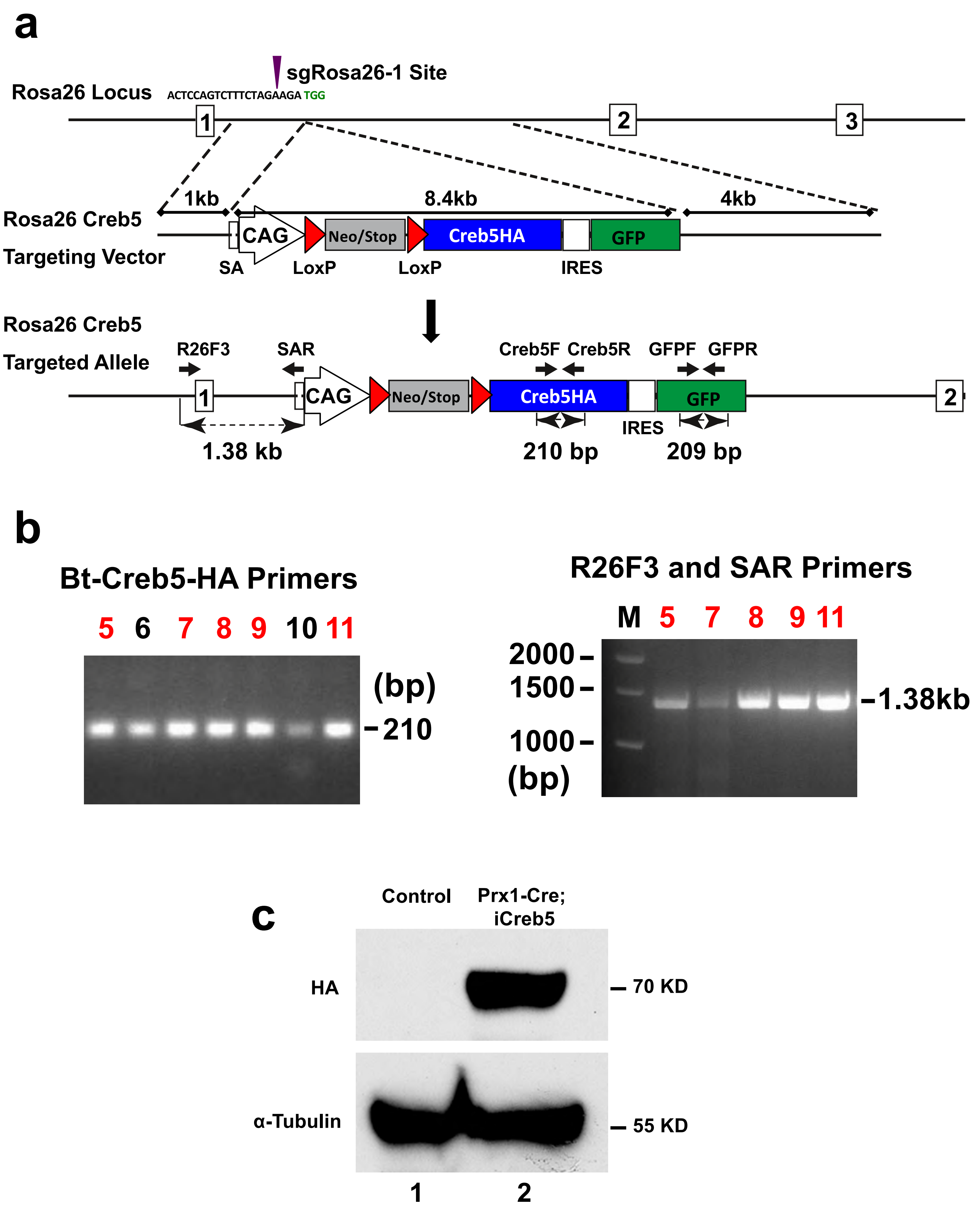

Supplementary Fig 4. Generation of *Prx1-Cre-iCreb5* mice. (a) WT *Rosa26* locus depicted with Cas9 sgRosa26-1 guide RNA; the Rosa26-iCreb5 targeting vector containing a CAG promoter, a loxP-flanked transcriptional termination cassette (LoxP-Neo/Stop-LoxP), and the bovine Creb5-HA cDNA linked to an ires-GFP reporter. (b) Diagnostic PCRs of tail DNA from some of the founder pups born after Cas9 and gRNA injection into one cell mice embryos (141 founder pups derived from these injected embryos were genotyped). Founder pups 5,7,8,9, and 11 contain both bovine Creb5-HA and GFP sequences, and also display homologous recombination of the targeting vector into the *Rosa26* locus as evidenced by generation of a 1.38 kb PCR-amplicon (with R26F3 and SAR primers). (c) Western analysis of Creb5-HA expression in limb tissue isolated from either a P0 *Prx1-Cre; Rosa26*$^{iCreb5\text{-}HA/+}$ (*Prx1-Cre-iCreb5*) mouse or a control littermate (that lacks either the *Prx1-Cre* or the *Rosa26*$^{iCreb5\text{-}HA}$ allele). Cre-mediated expression of transgenic iCreb5-HA was documented by both Western analysis (n=1 biological sample) and confirmed by immunostaining (n=5 independent biological samples) (some of which are displayed in Figs. 7c & 7e, and Supplementary Fig. 5b).

Supplementary Figure 5

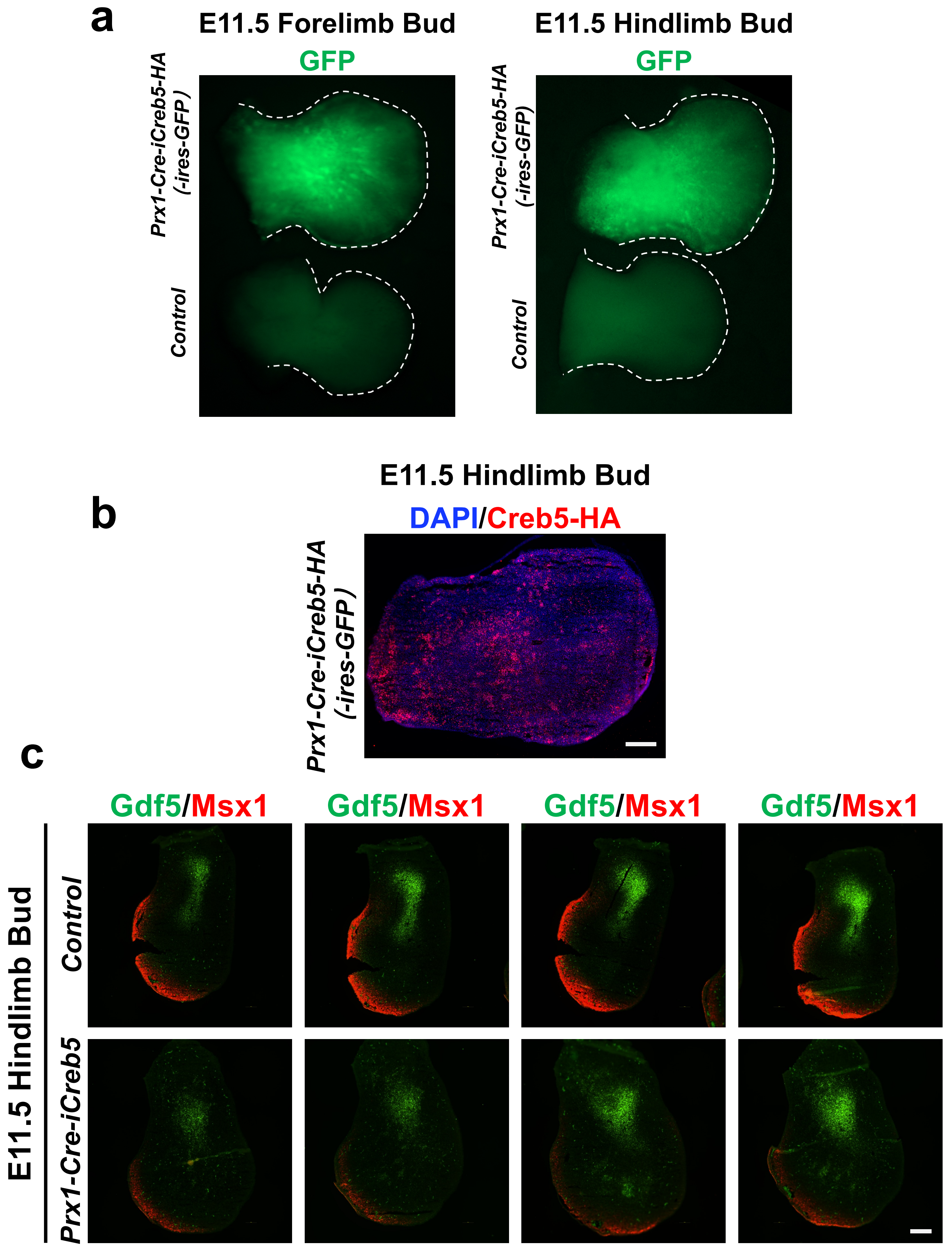

Supplementary Fig 5. Ectopic expression of iCreb5-HA-ires-GFP$^{(Prx1\text{-}Cre)}$ expands expression of Gdf5 and simultaneously decreases expression of the BMP-responsive gene Msx1 in E11.5 *Prx1-Cre-iCreb5* limb buds. (a) GFP whole mount fluorescence of limb buds of either E11.5 *Prx1-Cre-iCreb5HA-ires-GFP* embryos or their control littermates. Limb buds have been outlined with a dotted white line. Note the increased limb bud size in *Prx1-Cre-iCreb5HA-ires-GFP* embryos that express the iCreb5-HA-ires-GFP$^{(Prx1\text{-}Cre)}$ transgene. (b) Immunofluorescence detection of iCreb5-HA and DAPI in cryosection of the hindlimb of an in E11.5 *Prx1-Cre-iCreb5HA-ires-GFP* embryo. (c) RNAScope FISH in serial sections of a hindlimb bud of either a E11.5 *Prx1-Cre-iCreb5HA-ires-GFP* embryo or a control littermate. Similar results were obtained in 6 (a), 2 (b) or 5 (c) independent biological repeats. Scale bar equals 200 microns.

Supplementary Figure 6

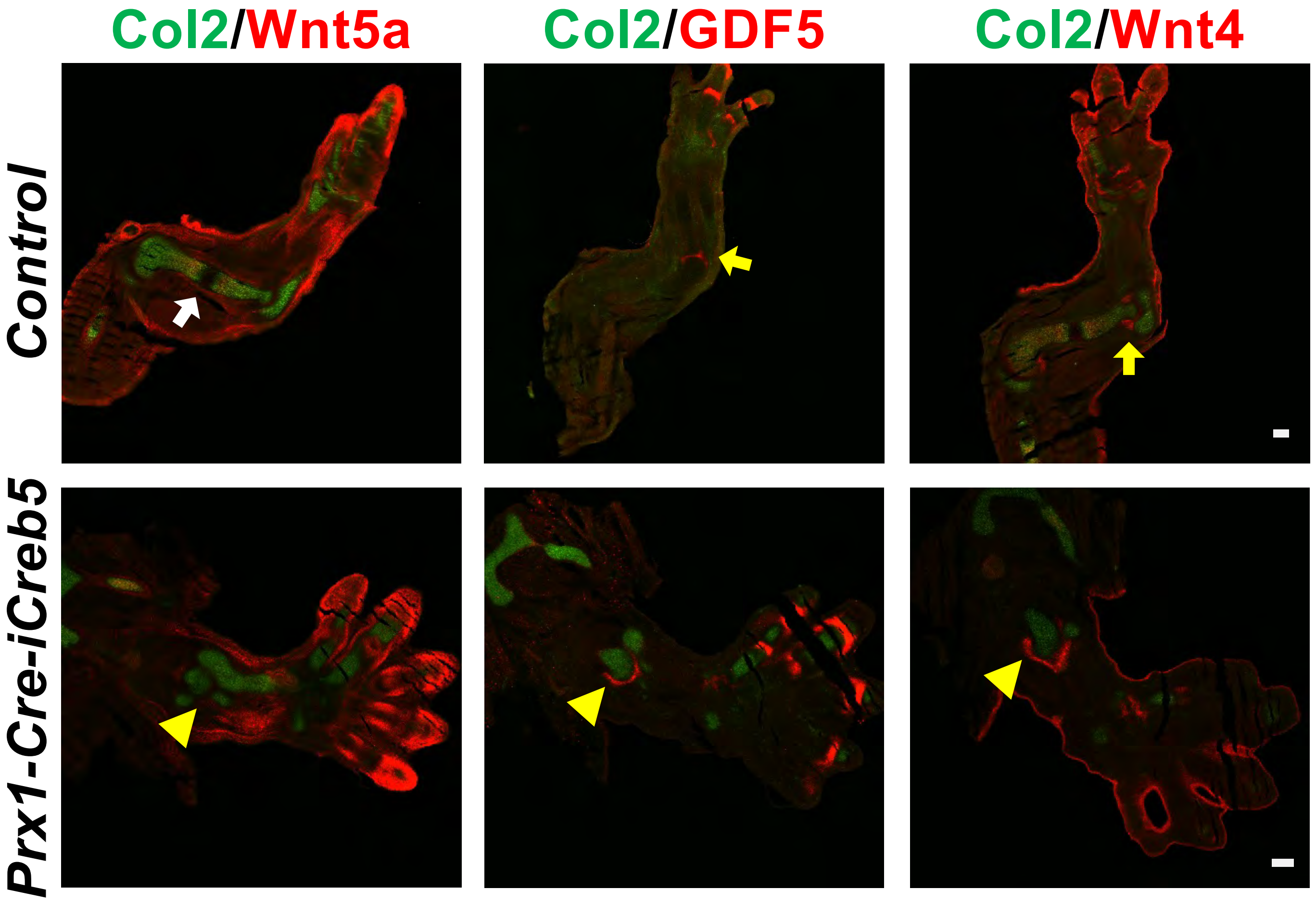

Supplementary Fig 6. Ectopic expression of iCreb5-HA$^{(Prx1-Cre)}$ can both boost the expression of interzone signaling molecules and repress expression of Wnt5a in the perichondrium. Gene expression assayed by FISH in serial sections of the forelimb of an E14.5 *Prx1-Cre-iCreb5* embryo or a control littermate. In control limbs, the articular perichondrium/joint interzone (yellow arrows) and metaphyseal/diaphyseal perichondrium (white arrows) are indicated. In *Prx1-Cre-iCreb5* limbs, the expression domain of the joint interzone markers Gdf5 and Wnt4 are expanded in the same area in which the Wnt5a^{+} perichondrium is absent (yellow arrowheads). Similar results were obtained in at least 2 independent biological repeats. Scale bar equals 200 microns.

Supplementary Figure 7

Control *Sox9iresCre-iCreb5* *Control* *Sox9iresCre-iCreb5*

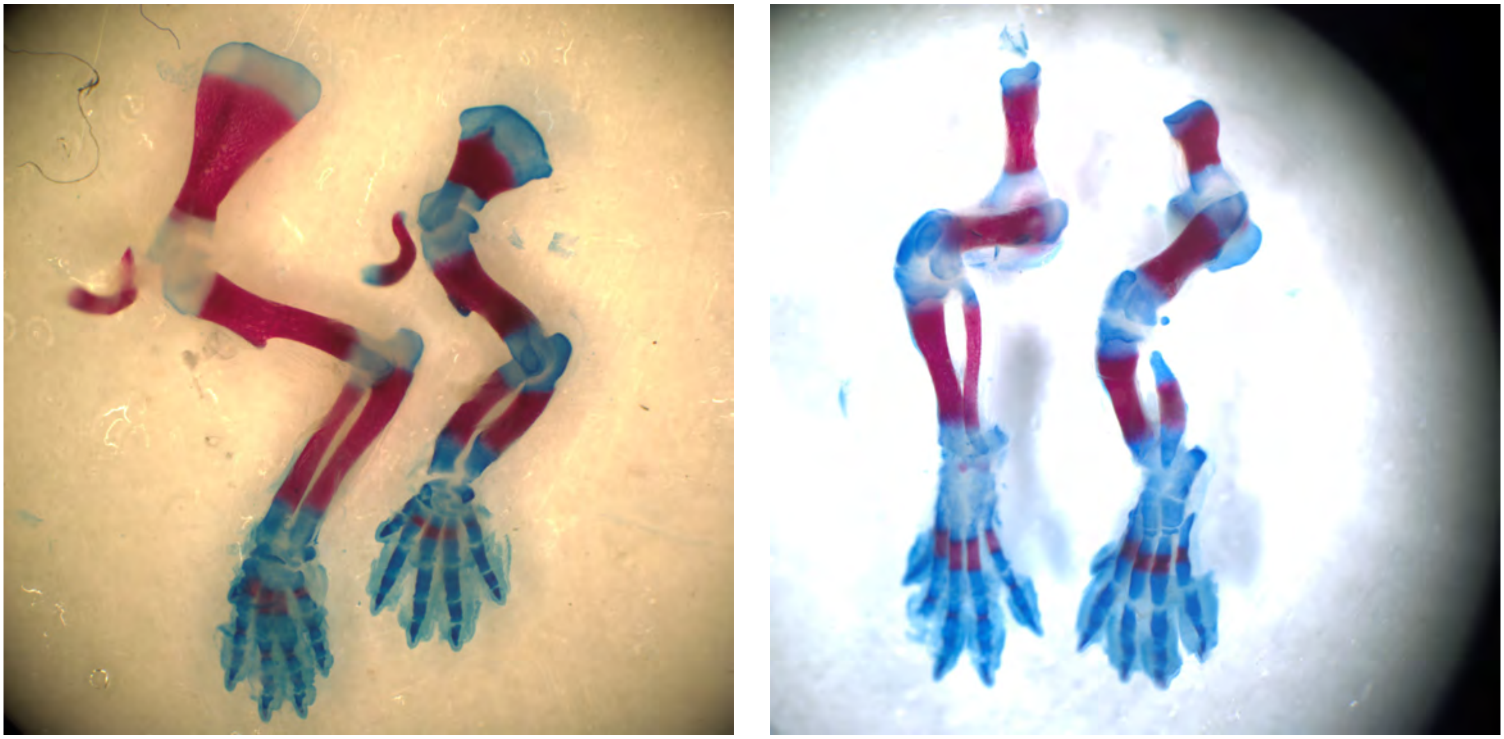

P0 Forelimbs **P0 Hindlimbs**

Supplementary Fig 7. Expression of iCreb5-HA$^{(Sox9\text{-}Cre)}$ in most chondrocytes and in a few perichondrial cells leads to shortened long bones in P0 *Sox9-ires-Cre-iCreb5* mice. Whole mount Alcian Blue/Alizarin Red staining of the appendicular skeleton of P0 *Sox9-ires-Cre-iCreb5* mice or their WT littermates. In addition to shortened long bones in P0 *Sox9-ires-Cre-iCreb5* mice, the deltoid tuberosity, olecranum, and patella are all present, but diminished in size. Similar results were obtained in 4 independent biological repeats.

Supplementary Figure 8

a

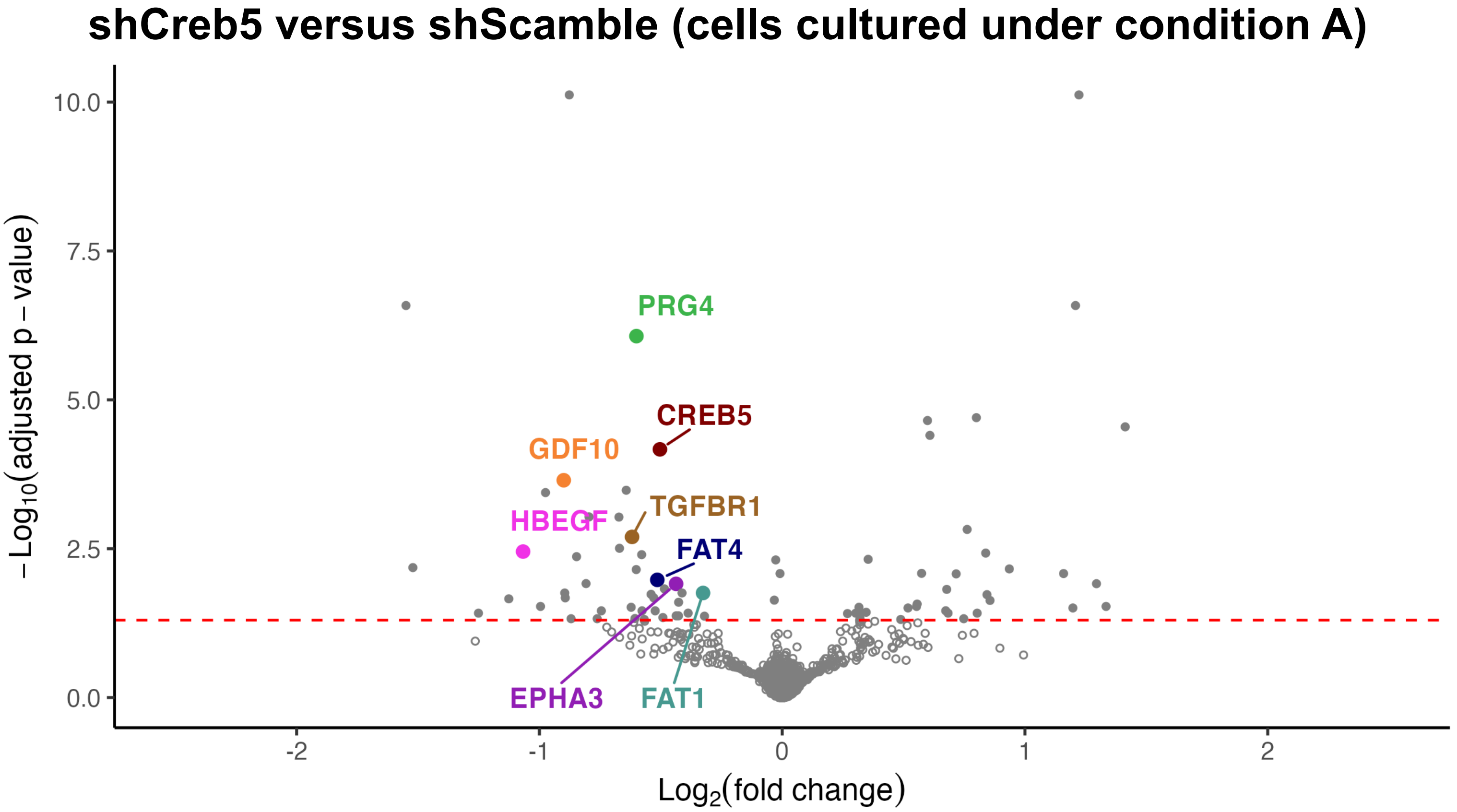

b

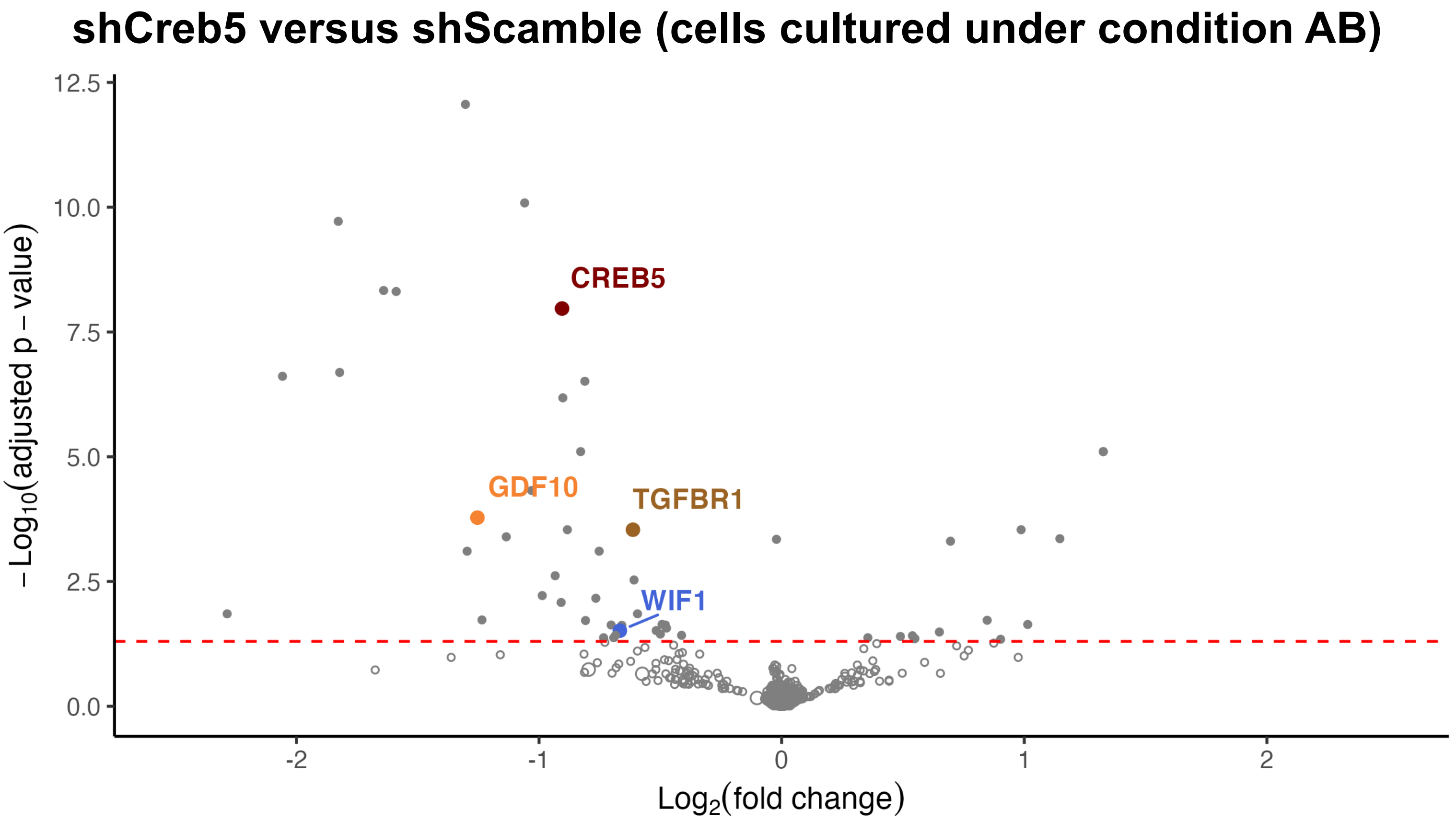

Supplementary Fig 8. Creb5 is necessary to maintain the expression of several classes of signaling molecules, including Wif1, Gdf10, Fat1, Fat4, EphA3, Hbegf (an EGFR-ligand) and Tgfbr1 in bovine superficial zone articular chondrocytes. We performed RNA-Seq analysis of bovine superficial zone articular chondrocytes that had been infected with lentivirus programmed to express either a control shRNA or shCreb5. Both TGF-β [31,32] and EGFR [78] signaling are necessary to maintain the expression of *Prg4* in the superficial zone of articular cartilage. Thus, bovine superficial zone articular chondrocytes that had been infected with lentivirus programmed to express either a control shRNA or shCreb5 were cultured in the presence of either the EGFR ligand, TGFα ("condition A"; a) or in the presence of both TGFα and TGFβ2 ("condition AB", b). RNA-Seq analysis was performed with n=4 independent biological repeats for each culture condition, and differentially expressed genes identified. The "Volcano" Graphs depicts the relative expression of genes whose expression was altered by knock-down of Creb5. Genes listed above the dotted red line display significantly differential gene expression (i.e., with an adjusted p value that is less than .05). Differentially expressed (DE) genes between treatment pairs were identified using DESeq2 [72] with FDR threshold of 0.05. The entire list of differentially expressed genes is detailed in Supplementary Data 1 and Supplementary Data 2.

Supplementary Figure 9

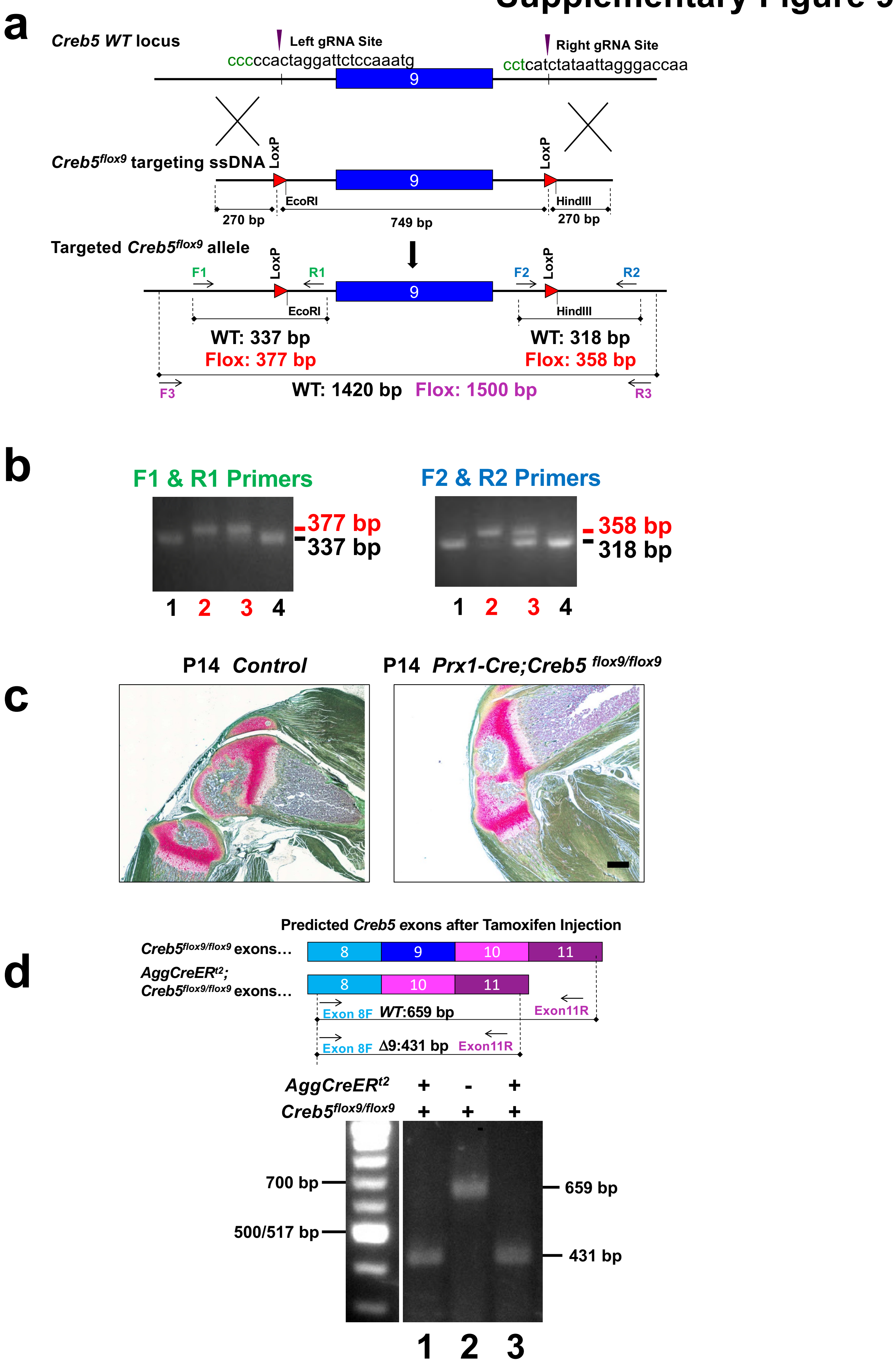

Supplementary Fig 9. Generation of the *Creb5^{flox9}* allele, which contains loxP sites flanking exon9 (which encodes the bZIP DNA binding domain) of *Creb5*. (a) WT *Creb5* exon 9 (blue) and flanking intron sequences depicted with targeting Cas9 left and right guide RNAs, and a loxP-flanked Creb5 single strand DNA as a recombination template. The targeted *Creb5^{flox9}* allele following homologous recombination is depicted. (b) Diagnostic PCRs of tail DNA (performed with primers depicted in (a)) from some of the founder pups born after injection of Cas9 protein, *Creb5^{flox9}* targeting single stranded DNA, and right and left gRNAs into one cell mouse embryos is displayed. 33 founder pups derived from these injected embryos were genotyped. Mice 2 and 3 (whose genomic DNA was amplified by the designated primers to yield both 377 bp and 358 bp PCR fragments) contain LoxP sites on both sides of exon9 of *Creb5*. Note that this PCR was performed with genomic DNA derived from the tails of founder *Creb5^{flox9}* mice. The tail of founder mouse 3 contains cells with either a *Creb5^{flox9}* allele or a WT allele. The *Creb5^{flox9}* allele in this animal successfully went germline, and thus we amplified the entire 1500bp sequence of the *Creb5^{flox}* allele (i.e., included in the F3 to R3 amplicon) from genomic DNA isolated from the F1 generation from founder mouse 3. This 1500bp sequence includes both LoxP sites that flank exon 9 and is displayed in Supplementary Table 6. (c) Safranin O/Fast Green staining of sections of the knee of either a P14 *Prx1-Cre; Creb5$^{flox9/flox9}$* female mouse or a *Creb5$^{flox9/flox9}$* male littermate (control). Scale bar equals 500 microns. Note that the *Prx1-Cre; Creb5$^{flox9/flox9}$* mouse displays fusion of the knee joint, which phenocopies the fusion of the knee joints observed in *Creb5$^{\Delta9/\Delta9}$* mice. A similar phenotype was observed in n=5 independent *Prx1-Cre; Creb5$^{flox9/flox9}$* animals. (d) *Creb5$^{flox9/flox9}$* mice were mated with *Aggrecan1$^{tm(IRES-CreERT2}$;Creb5$^{flox9/flox9}$* mice to generate litters containing both *Aggrecan1$^{tm(IRES-CreERT2)}$; Creb5$^{flox9/flox9}$* mice or *Creb5$^{flox9/flox9}$* control mice (which lack the CreERt2 driver allele). Tamoxifen was repeatedly administered to these litters at postnatal days 1-11, to specifically delete exon 9 of *Creb5$^{flox9/flox9}$* in postnatal stage articular chondrocytes. Following sacrifice at P14, RNA was isolated from the femoral heads of either *Aggrecan1$^{tm(IRES-CreERT2}$;Creb5$^{flox9/flox9}$* mice (lanes 1

& 3) *or Creb5*$^{flox9/flox9}$ mice (lane 2). The retention or absence of *Creb5* exon 9 in Creb5 transcripts was assayed by RT-qPCR with primers located in *Creb5* exon 8 (Exon 8F: ACATGATGGAGATGATGGGCTC) and in *Creb5* exon 11 (Exon 11R: GTGTTGGATAACTTGCTGCTGA). Note that exon9 of *Creb5*$^{flox9/flox9}$ was deleted in femoral head articular cartilage specifically in the tamoxifen treated *Aggrecan1*$^{tm(IRES\text{-}CreERT2}$*;Creb5*$^{flox9/flox9}$ mice (lanes 1 & 3). Similar results were obtained in femoral caps isolated from n=4 different tamoxifen treated *Aggrecan1*$^{tm(IRES\text{-}CreERT2}$*;Creb5*$^{flox9/flox9}$ mice.

Supplementary Figure 10

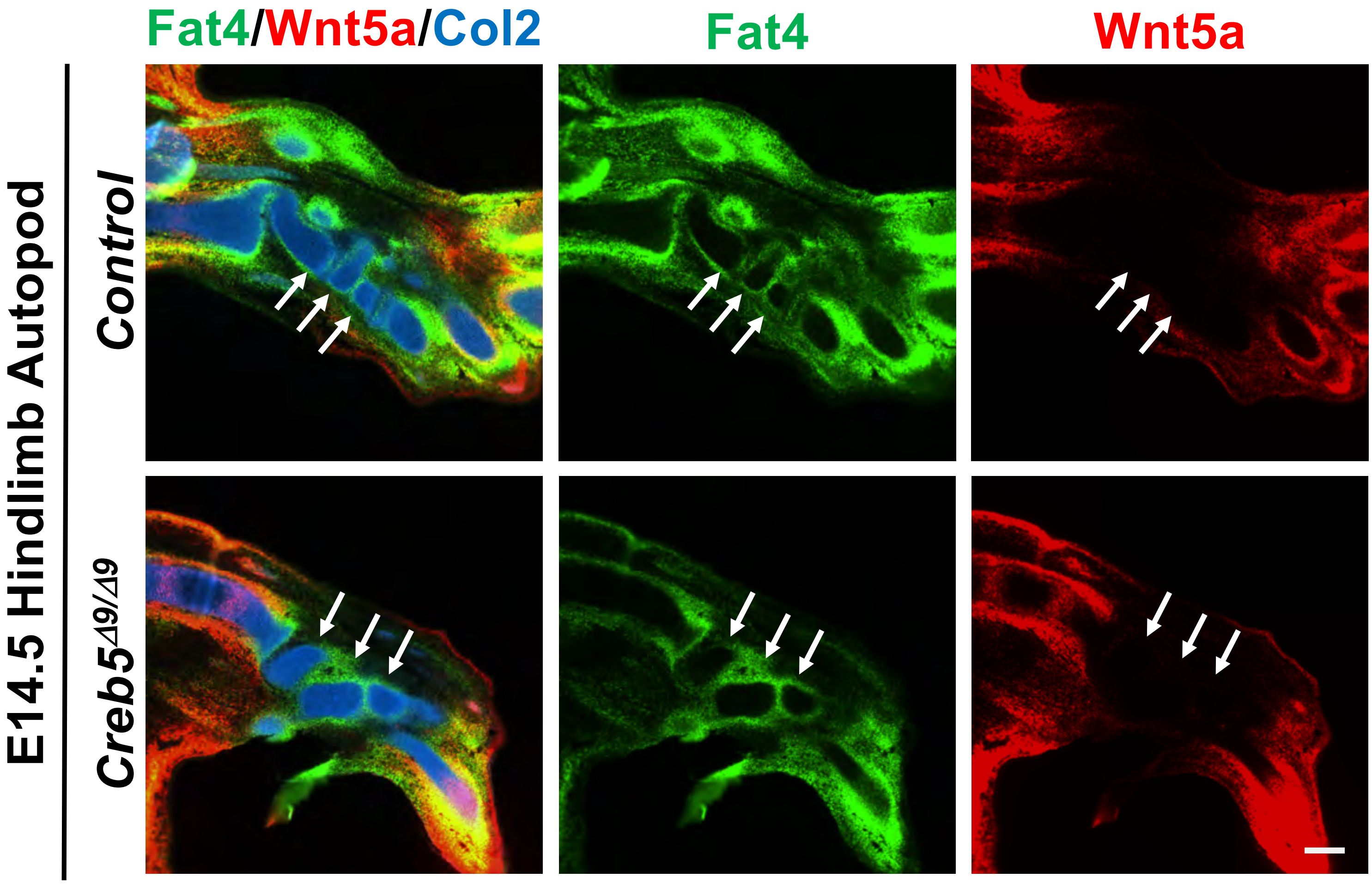

Supplementary Fig 10. The mesopodial skeleton joints that still form in *Creb5*$^{\Delta 9/\Delta 9}$ mice lack Wnt5a expression in their interzones. Gene expression assayed by RNAscope FISH in the hindlimb autopod of an E14.5 *Creb5*$^{\Delta 9/\Delta 9}$ embryo or a control littermate. Arrows indicate the location of the Fat4^{+}/Wnt5a^{-} perichondrium surrounding the mesopodial cartilage elements. Similar results were obtained in 3 independent biological repeats. Scale bar equals 200 microns.

Supplementary Figure 11

Uncropped gel images of the indicated figures (page 1 of 7)

Supplementary Figure 1b

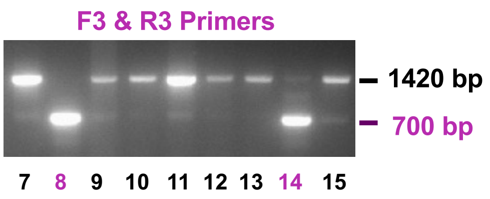

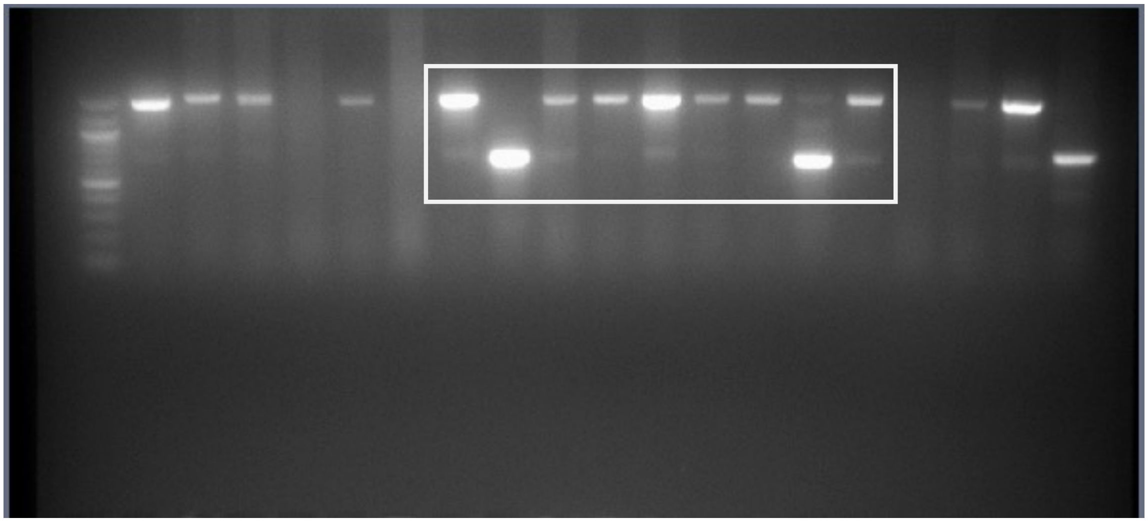

Supplementary Figure 1c

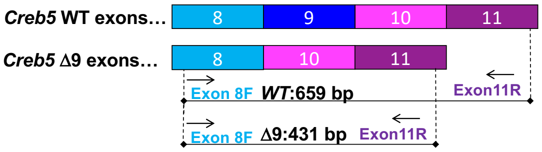

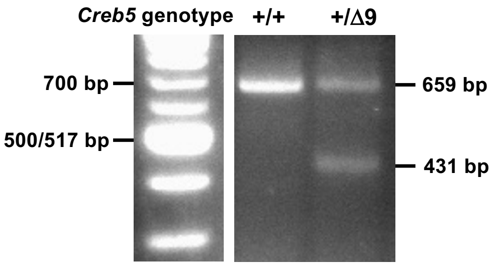

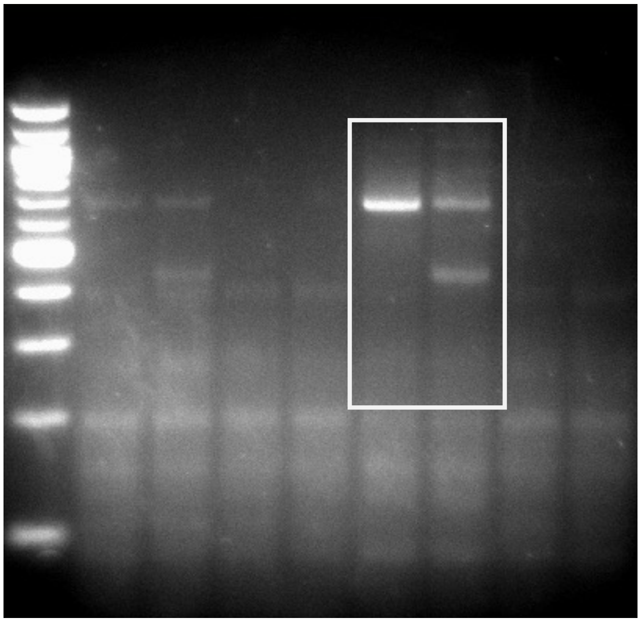

Uncropped blot images of the indicated figures (page 2 of 7)

Supplementary Figure 1e

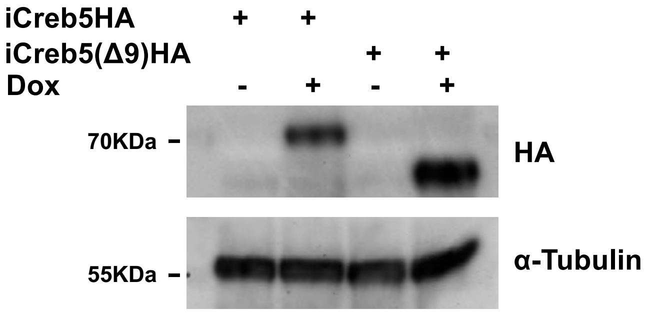

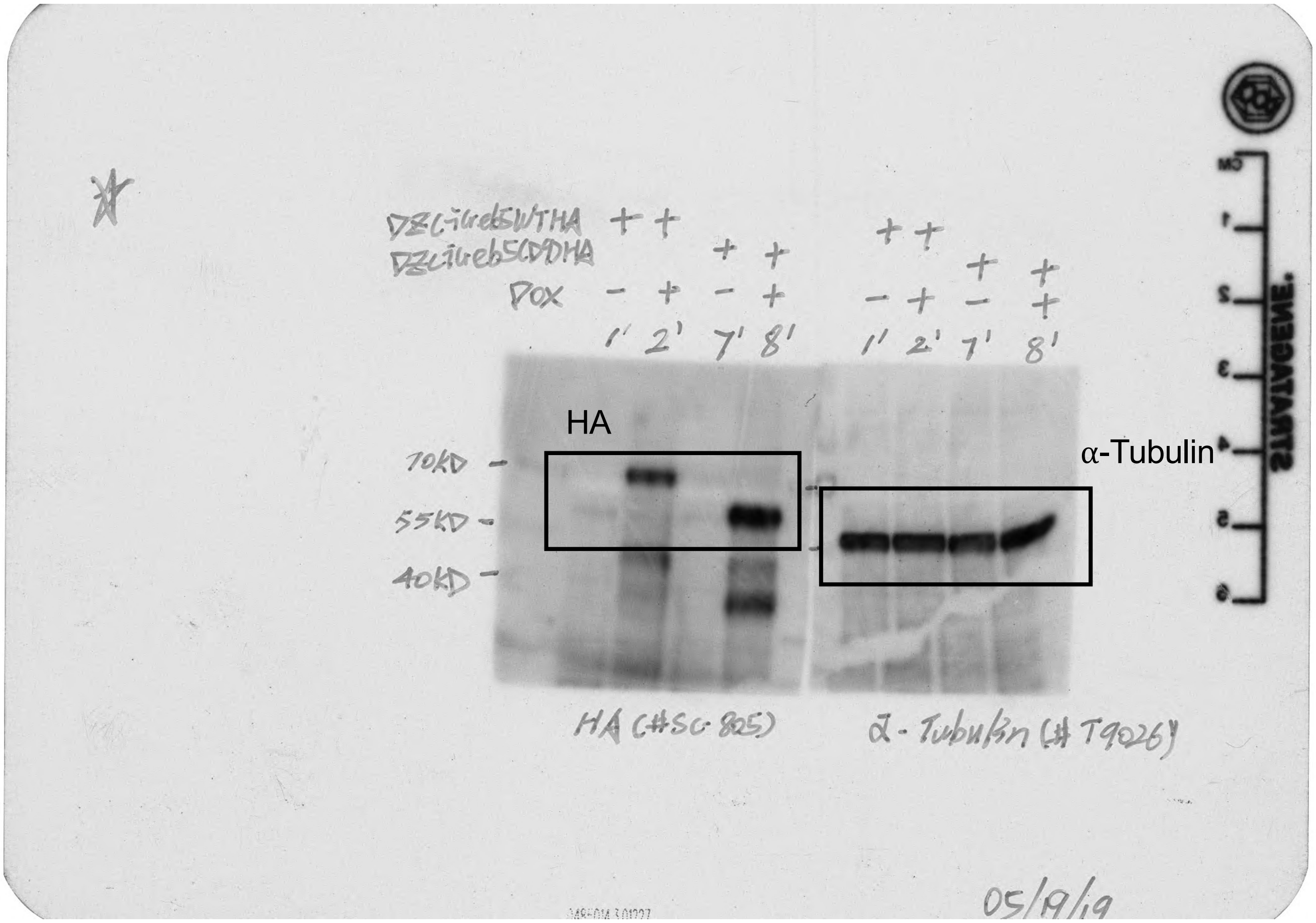

Uncropped gel images of the indicated figures (page 3 of 7)

Supplementary Figure 4b

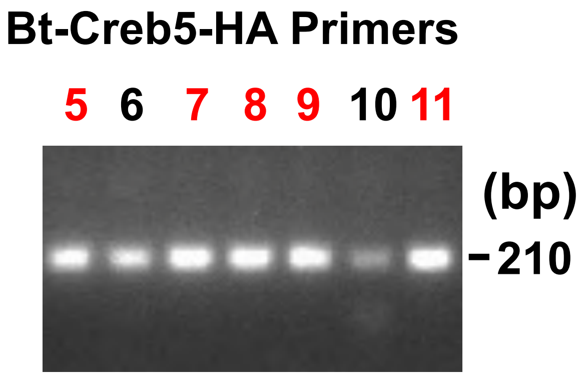

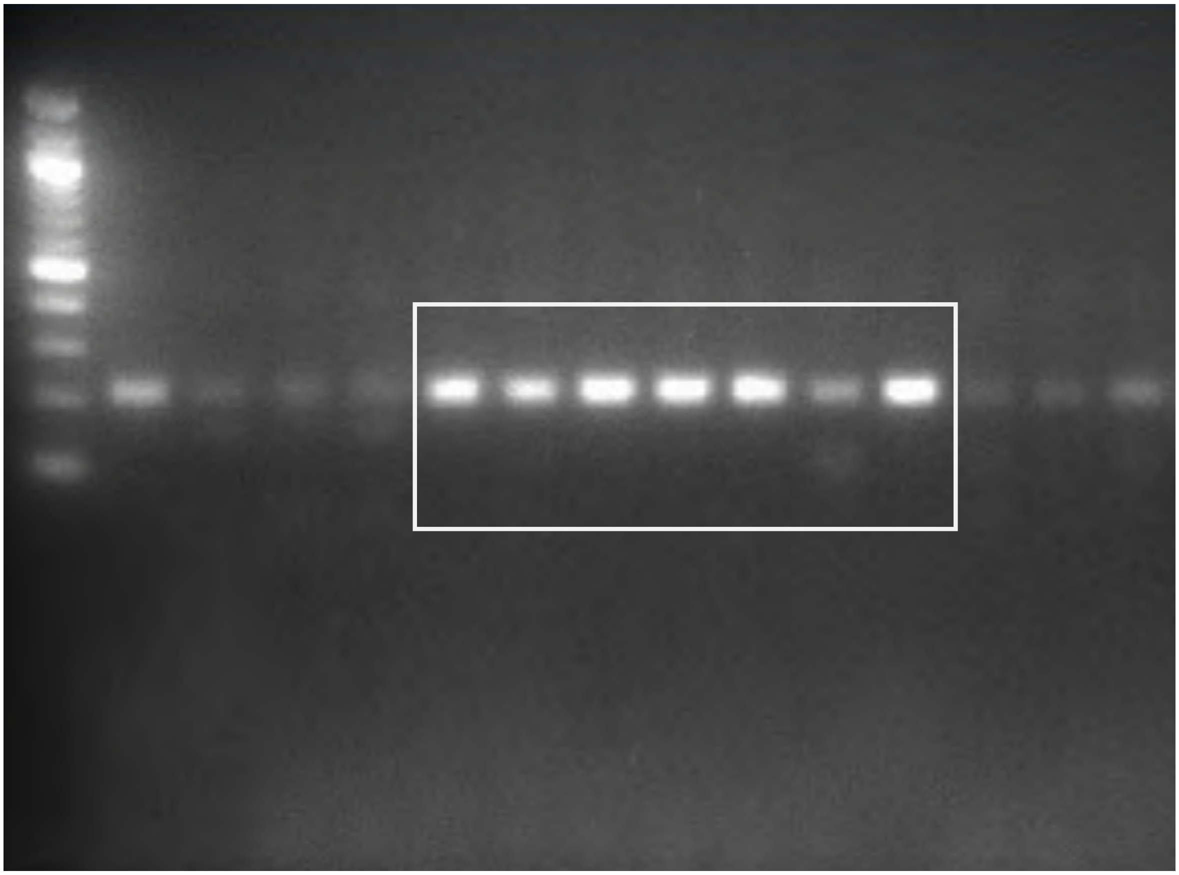

Uncropped gel images of the indicated figures (page 4 of 7)

Supplementary Figure 4b

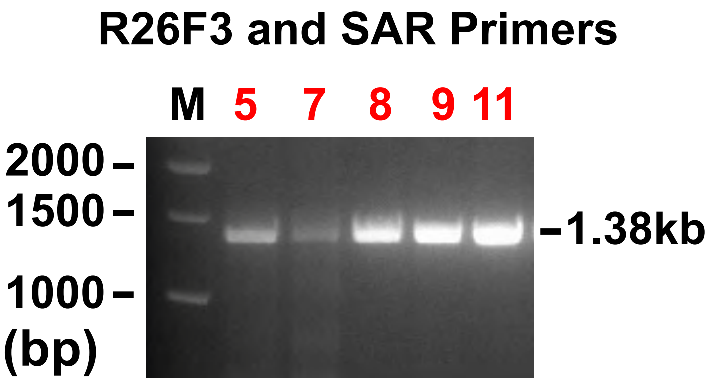

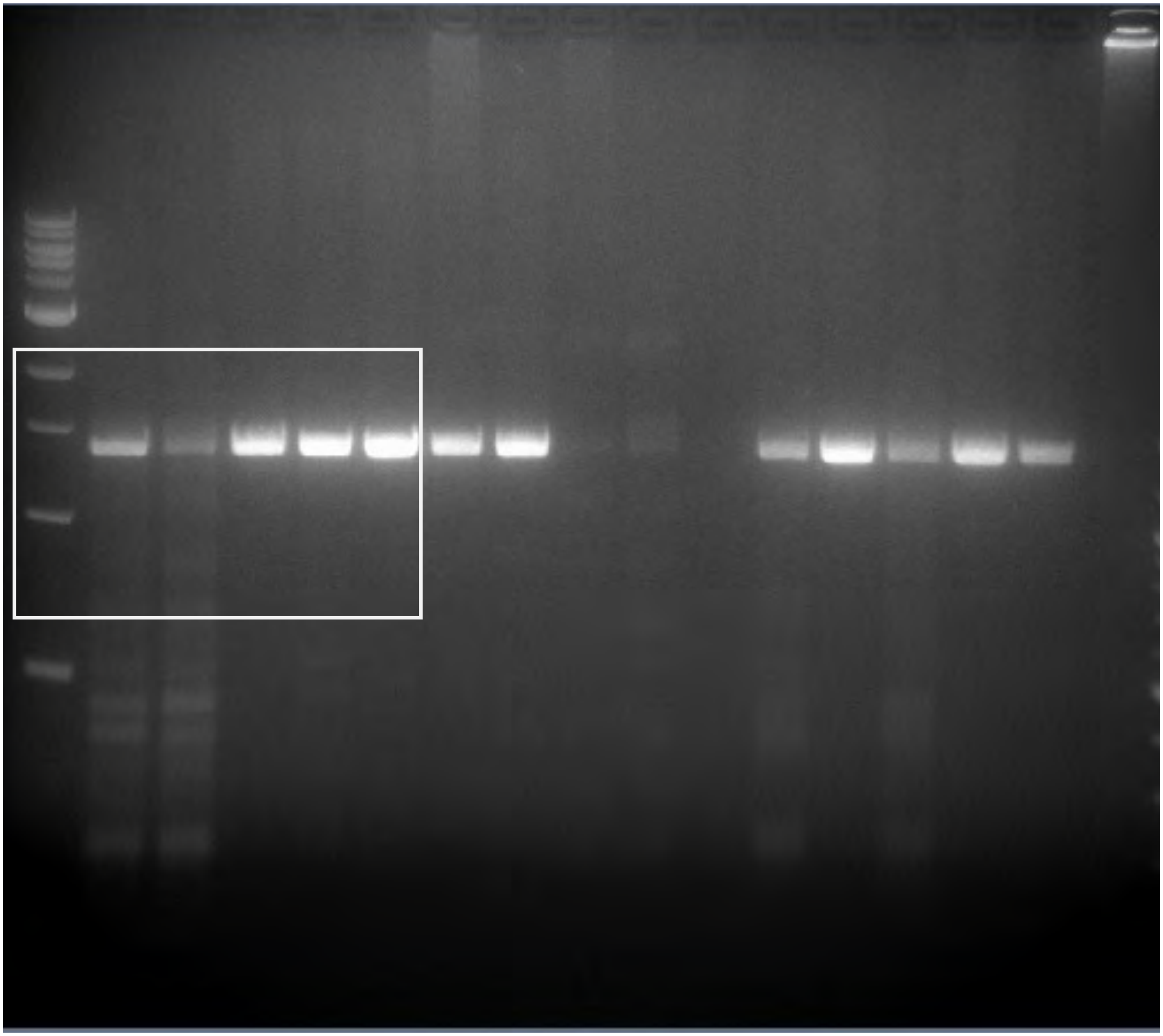

Uncropped blot images of the indicated figures (page 5 of 7)

Supplementary Figure 4c

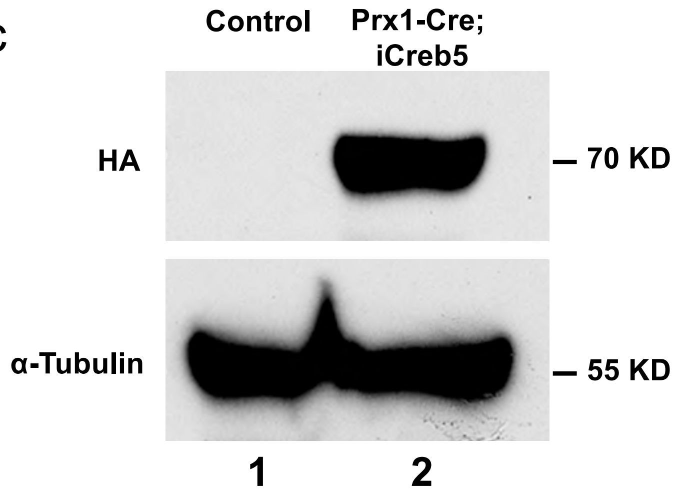

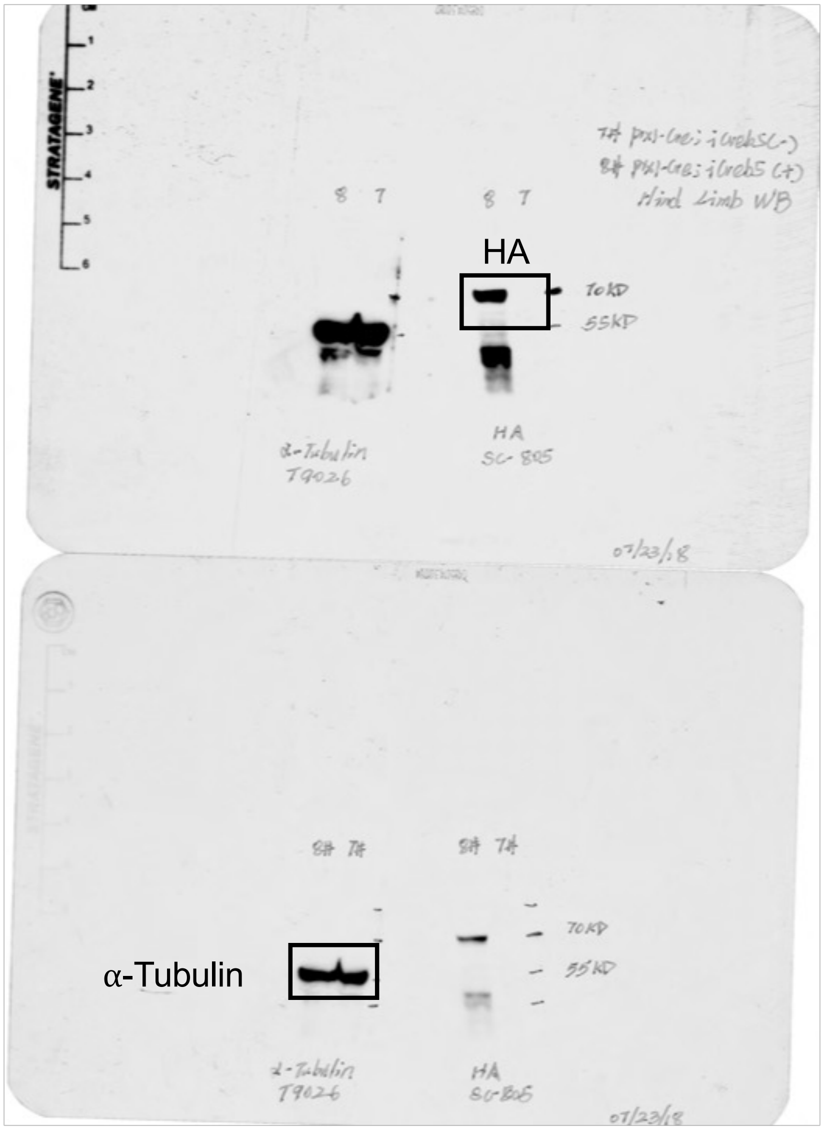

Uncropped gel images of the indicated figures (page 6 of 7)

Supplementary Figure 9b

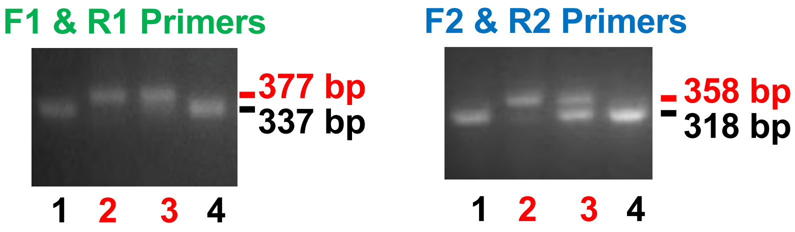

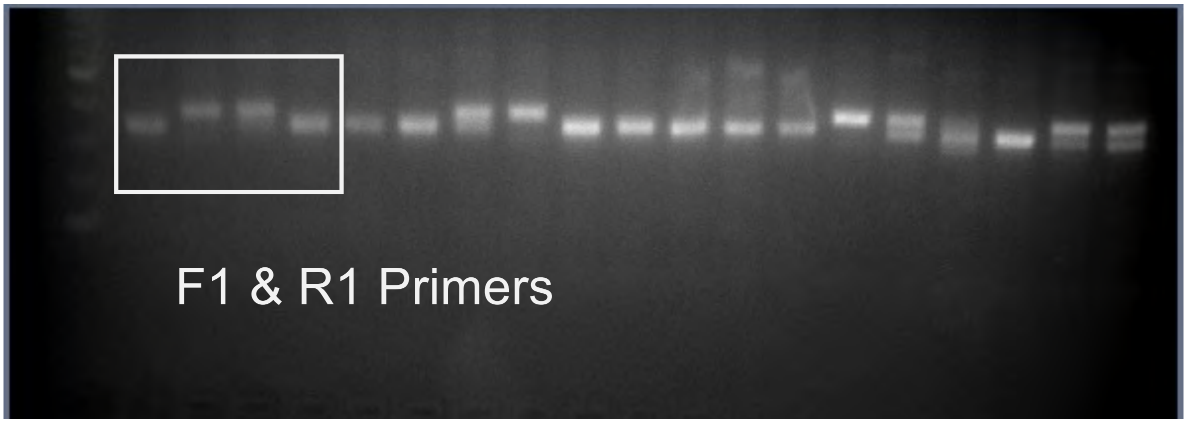

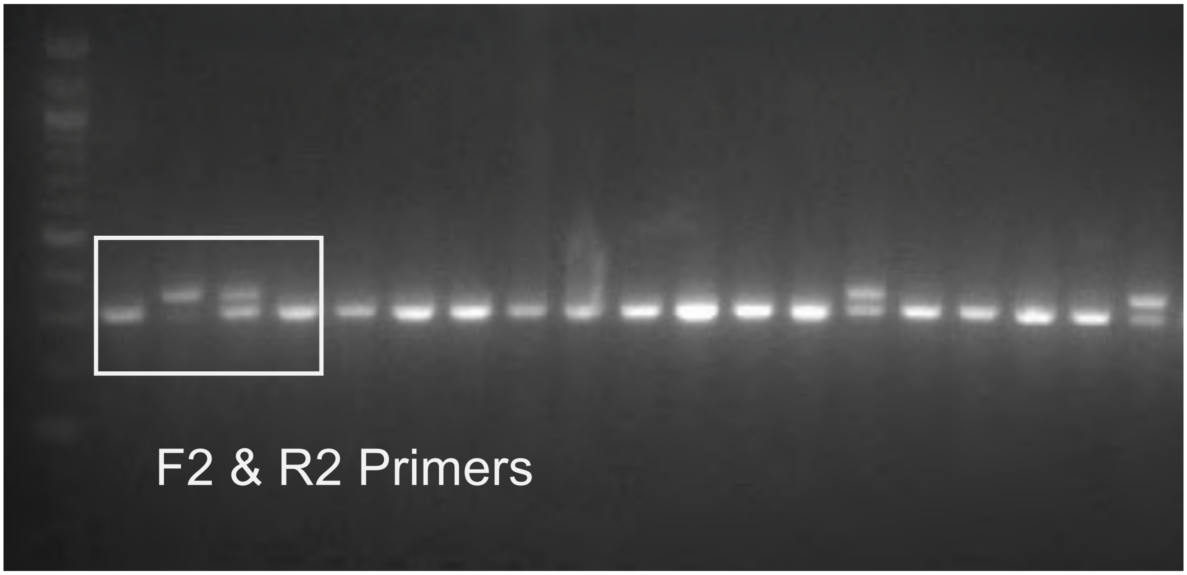

Supplementary Figure 11

Uncropped gel images of the indicated figures (page 7 of 7)

Supplementary Figure 9d

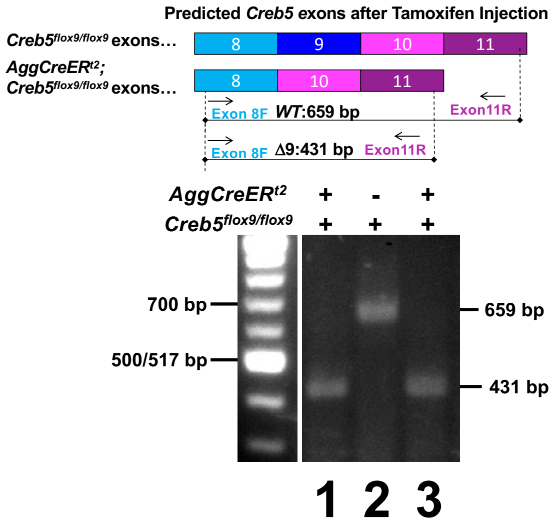

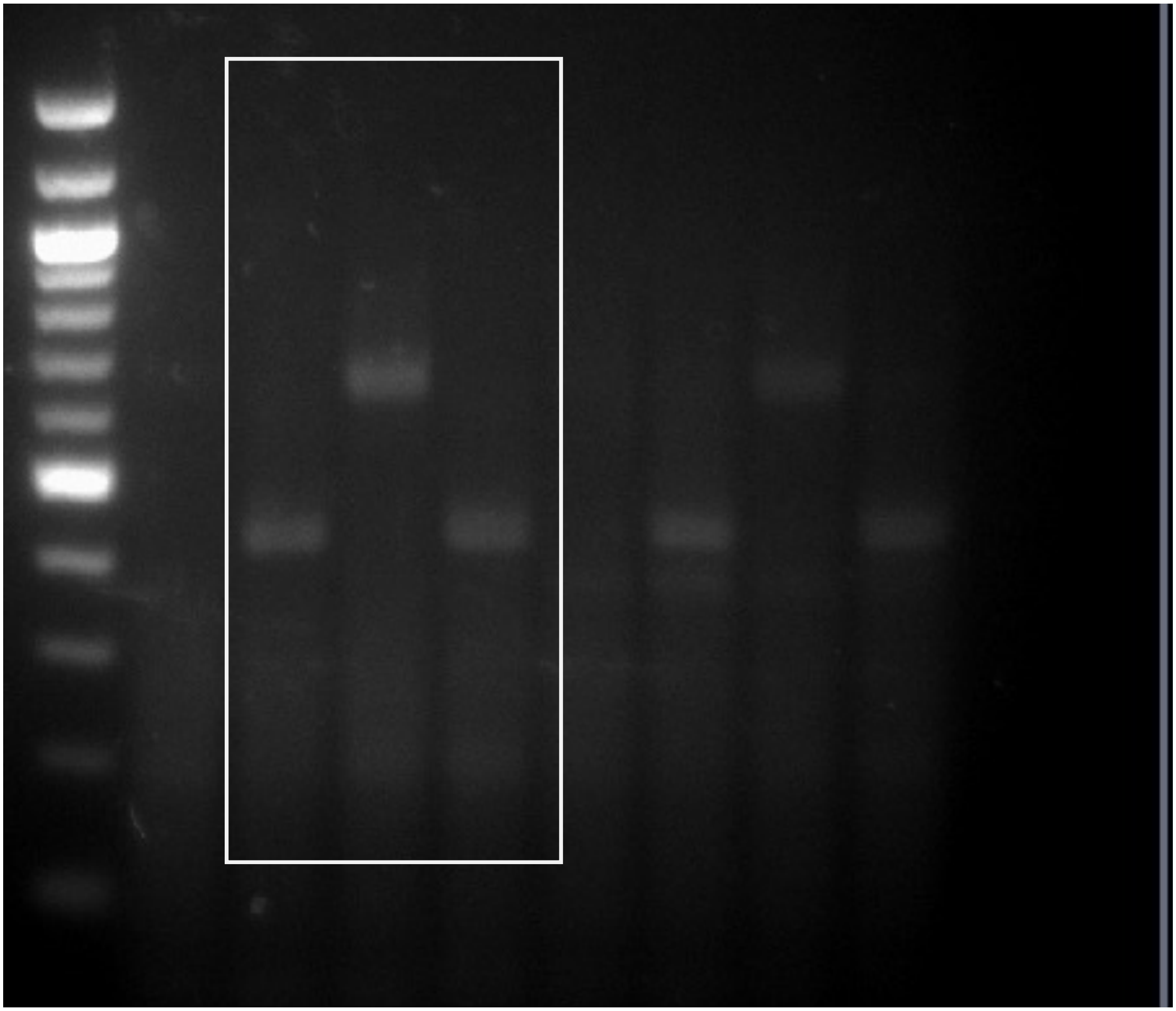

Supplementary Fig 11. Uncropped images of the indicated Figures.

Supplementary Tables

Supplementary Table 1 : RT-qPCR Primers to amplify bovine cDNA

Gene	Forward	Reverse
Prg4	CTTGATTCAGCAAGCTTCTTCTC	ACAGGAAAGCTCCACAGTGC
Gapdh	TGGTGAAGGTCGGAGTGAAC	TGTAGACCATGTAGTGAAGGTCA
Creb5	CCA ACC ATG ACA CCA GCG	GCA TGG GCT GTC TCT GTC

Supplementary Table 2: Antibodies employed for Western Blots

Cat. No.	Concentration	Target	Host	Vendor
T9026	1:1000	α-Tubulin	Mouse	Sigma
sc-805	1:1000	HA	Rabbit	Santa cruz biotechnology

Supplementary Table 3: Antibodies employed for immunohistochemistry

Cat. No.	Concentration	Target	Host	Vendor
PA5-65593	1:100	Creb5	Rabbit	Thermo Fisher
11867423001	1:100	HA	Rat	Sigma
3724S	1:200	HA	Rabbit	Cell Signaling Technology
AB5535	1:100	Sox9	Rabbit	EMD Millipore
18338S	1:50	Phospho-Smad2 (Ser465/Ser467)	Rabbit	Cell Signaling Technology
A11029	1:250	anti-Mouse IgG; Alexa Fluor 488	Goat	Thermo Fisher
A11037	1:250	anti-Rabbit IgG ; Alexa Fluor 594	Goat	Thermo Fisher
A11006	1:250	anti-Rat IgG; Alexa Fluor 488	Goat	Thermo Fisher
S11227	1:250	Streptavidin, Alexa Fluor™ 594 conjugate		Thermo Fisher

Supplementary Table 4: Growth factors and inhibitors used for chondrocyte culture

	Cat. No.	Concentration	Vendor
**TGF-α**	**239-A-100**	**100 ng/ml**	**R&D Systems**
**TGF-β2**	**302-B2-010**	**20 ng/ml**	**R&D Systems**
**Doxycycline**	**D9891-1G**	**1 μg/ml**	**Sigma**

Supplementary Table 5: Target sequence (sense strand) of in situ hybridization probes.

**Prg4**	AGATTCAGAACAAACCTGAAGAAACAACTCCTGCATCAGAAGATTCTGAT<br>GATTCTAAAACAACTCTAAAACCACAGAAGCCAACCAAAGCACCCAAGCC<br>TACCAAAAAGCCAACCAAAGCACCCAAGAAGCCCACCTCTACCAAAAAGC<br>CAAAGACACCAAAAACAAGAAAACCAAAAACTACACCAGCTCCTCTAAAG<br>ACGACTTCAGCAACACCTGAACTGAATACCACCCCTCTAGAAGTCATGCT<br>GCCAACCACCACCATCCCTAAACAAACTCCAAACCCTGAAACAGCTGAAG<br>TAAATCCAGATCATGAAGATGCAGATGGAGGTGAAGGAGAAAAACCTCT<br>GATTCCCGGGCCCCCTGTGCTATTCCCCACAGCTATTCCAGGCACTGATC<br>TTTTGGCCGGGAGACTCAATCGAGGCATTAACATCAATCCCATGCCTTCA<br>GATGAGACCAATTTATGCAATGGTAAGCCAGTGGATGGACTGACTACGCT<br>GCGCAATGGGACATTAGTTGCATTTCGAGGTCATTATTTCTGGATGCTGA<br>ATC
**Murine Creb5**	GCCTGTCCCAGGCTCTCTATCATCTCTACTCCATCTCCACAACAGACAGAG<br>GCAGCCCATGCCGGCCTCCATGCCTGGAACCCTGCCCAACCCCACCATGC<br>CAGGATCTTCTGCCGTCTTGATGCCTATGGAGAGACAGATGTCAGTGAAC<br>TCCAGCATCATGGGCATGCAAGGTCCAAACCTCAGCAACCCCTGTGCTTC<br>TCCCCAAGTCCAGCCAATGCATTCAGAAGCCAAAATGAGACTGAAGGCTG<br>CGCTGACTCACCATCCTGCCGCCATGTCGAACGGGAACATGAGCACCATC<br>GGACACATGATGGAGATGATGGGCTCCCGGCAAGACCAGACACCGCACC<br>ACCACCTGCACTCACACCCGCATCAGCACCAGACACTGCCGCCCCACCAC<br>CCCTACCCACACCAGCACCAGCACCCCGCACACCATCCCCACCCACAGCCT<br>CACCACCAGCAGAACCACCCGCACCACCACTCCCATTCCCACCTTCACGCA<br>CACCCGGCGCACCACCAGACCTCGCCACACCCACCCCTGCACACCGGCAA<br>CCAAGCACAGGTTTCACCAGCTACACAACAGATGCAGCCAACCCAGACAA<br>TACAACCACCCCAGCCCACAGGGGGACGCCGGCGAAGAGTGGTGGATGA<br>GGACCCAGATGAGAGGCGGAGGAAATTTCTGGAAAGGAACCGGGCCGCC<br>GCCACCCGCTGCAGACAGAAGAGGAAGGTCTGGGTGATGTCACTGGAAA<br>AGAAAGCTGAAGAGCTCACCCAGACAAACATGCAGCTTCAGAATGAAGT<br>GTCCATGTTGAAAAACGAGGTGGCCCAGCTGAAGCAGTTGTTGTTAACAC<br>ATAAAGACTGTCCGATAACAGCCATGCAGAAAGAATCCCAAGGGTATTTA<br>AGTCCAGAGAGCAGCCCTCCTGCGAGCCCTGTGCCAGCATGCTCTCAGCA<br>GCAAGTTATCCAACACAACACCATCACTACATCCTCATCGGTCAGCGAGG

	TCGTGGGGAGCTCCACCCTCAGTCAGCTTACAACTCACAGAACAGACCTG<br>AATCCTATTCTTTAAAAGGCATCGGTCAAACCTGGCCTTTGAGAAGAGCT<br>GTAGCATGCCGTACATCCTTTCTCAAAGGGGCATTTTTTTTAGAATTATCT<br>CAGACCTGGAAGACGCCTCAGCCCTTCAAAGACTGGCTTTCATTTTTATA<br>GTTATTATGGAAATGTTGTCTTTTATACTTAGTTATATAAGAAAAAAGGG<br>AGATATGCAATGAATATCTATCAGCTTGGGGAGCACGTTGGTGCTTCTCT<br>GCAATTTTCTGGTACCAGTTTCTTGTTTATAAACGGAACCTTTCCTGTATA<br>TAGCCATGGTTTCATTCTTACCAGCCCAACCCTTTGCCTGGAACAATGAAT<br>CTTGTTCAACTACAGCTTTTAGCCAAAATGAGGTATGCTTAGATGTCAAG<br>CGAGATGGATCCACACAGTAACTGGGTGGGAAAGCTCATGATGTCATAA<br>CTCATGTTGAGTTTGTGCTGTGATGTCACCAGAATCTCAGATAAACACAT<br>GGGCCTTCCTGAATATTTTTTCTCTTGCTAGAAAAAAATAAATTATGGTCC<br>ATCCATATCCCATGAAAGCCACCAAGCATCTCAGGCCCCCTCCTTCCTCTC<br>TTTTCTACTTGTGCAGATGTCCAATATCCATCTCATTTTCTTTTCCCCGGA<br>TCCCTTGTTTACTCTTTGTTTTGACTTTTTTTCTGTTTCCTTTTCTCCCCTT<br>TAGCTTTGCATGTAAAAAAGAAAATAATGTTTAAAGAAGAGAAAAAGCAA<br>ATCTGGAAACTGTGGACCTAGCCACAGTTTAACCCACAGCTGGAGTTCAT<br>TCAATTTTTGCCTTTCACAAAATAGCAACCAGGAGATGTTTAATGTGCCT<br>GATTTAATGTTTTTAATAACCAGAGCAAATAAAAGGTGGTTTGGTTATAG<br>GTGAAGCACTGTTGAATGCCAGCTGTGGGGACACTAGGGAAAGGGACTT<br>CGTAAGCTCCAACTGTGAAAATTCAAATAAGGATGTGGGCTCTAACATCA<br>CACCCTCGAATTACAGCTCGCTTCTATGGCCTGTCTATAATGTAAAAAATC<br>CATGCACTATATAATAGTTCAGAAGGGCTCTGTTCACTACACAGATTACA<br>TTGTTCAATCATCAGCTGCTAATAACCTAAGATTTATTATTATTATTTTTC<br>TTAAGCCTATGGAACCAGCTCTGCTGTTCTGGTGGGCAAAAGCAAACTCA<br>CTCTTGGAGCAACAGAGAGAAAGCGAGGCCAGCGTTTCTCGGGGACTCG<br>CAGTCTGCCAGAACAGTCAGACTCCTTGGCTGCTGACCGAGTCCCATGGA<br>GGTGGCCAGGCTGGTGCGCTCATCTGAGTAGTTCTGATTTATATTTTCAG<br>CAATGTCCACGGACTTGCCCATTACAGAAAGCAGATCAAACCCAAACCAC<br>AGTTGTGCCTCCTTGAAACAAGCCATTCTACTCTGCTGGTGTTTTACTATC<br>GTGTTTCACAAATAATAGGGGCTAATGTTTCTCACTAGCAGTCTGGGCAT<br>ATGCTGGTGTTTCATCTCTGCCCAAATAATTCACCTCCTAACCTATGTGTG<br>TGTGTGTGTGCACATGGATGTGTGTGCCTGAGTGTGTGAGTGTGTGTGT<br>GTGTGTGTGTGTGTGTGTGTGTGTTTATGAACAGTATAGGTTTTAAAAGA<br>ACAGTATTTTACAAAAGCCATCACTTTTATAAGAGTTCTGTAAAGGAAGG<br>ATGTACTTCTTCGCTCACTATAGTTTAAAAAAATTCTATTTTAGAGGAAAA<br>AAAAAAAAAAAAAAAA
**Col2a1**	CCTGTCTGCTTCTTGTAAAACCCCCGAACCCTGAAACAACACAATCCATTG<br>CGAACCCAAAGGACCCAAACACTTTCCAACCGCAGTCACTCCAGGATCTG<br>CACTGAATGGCTGACCTGACCTGATGATACCCAACCGTCCTCCCCTCACA<br>GCCCGGACTGTGCTCCCCTTTCTAAGAGACCTGAACTGGGCAGACTGCAA<br>AATAAAATCTCGGTGTTCTATTTATTTATTGTCTTCCTGTAAGACCTCTGG<br>GTCCAGGCGGAGACAGGAACTATCTGGTGTGAGTCAGACGCCCCCCGAG<br>TGACTGTTCCCAGCCCAGCCAGAAGACCCCTACAGATGCTGGGCGCAGG<br>GACTGCGTGTCCTACACAATGGTGCTATTCTGTGTCAAACACCTCTGTAT<br>TTTTTA
**Matrilin1**	GGATCCAAGAGCGTGCGGCCTGAGAACTTTGAGCTGGTGAAGAAGTTCA<br>TCAACCAGATTGTGGACACGTTAGATGTGTCGGACAGGCTAGCCCAGGT<br>GGGGCTGGTGCAGTACTCCAGCTCCATTCGCCAGGAGTTCCCACTCGGCC<br>GCTTCCACACCAAGAAGGACATTAAGGCCGCGGTGCGGAACATGTCCTAC<br>ATGGAGAAAGGCACCATGACTGGCGCCGCCTTGAAGTATCTCATAGATAA

	TTCTTTCACTGTGTCCAGCGGGGCAAGGCCTGGAGCCCAGAAGGTGGGC<br>ATCGTCTTCACCGATGGCCGGAGCCAGGACTACATTAATGACGCTGCCAG<br>GAAGGCCAAGGACCTTGGCTTTAAGATGTTTGCGGTGGGCGTGGGCAAT<br>GCTGTGGAGGAAGAGCTGAGGGAGATCGCTTCCGAGCCCGTGGCAGACC<br>ACTACTTTTACACAGCTGACTTCAAGACCATCAACCAGATTGGCAAGAAG<br>CTGCAGAAACAAATCTGTGTGGAGGAAGACCCCTGTGCTTGTGAGTCCAT<br>ACTGAAATTTGAGGCCAAGGTGGAGGGTCTGCTGCAGGCCCTGACCAGG<br>AAGCTGGAAGCTGTGAGCGGGCGGCTGGCTGTCCTGGAGAACAGAATCA<br>TCTAA
**Wif1**	CCCACGCGTCCGCCCACGCGTCCGGTGGAGGGAAGGTTAAGGACTGTGG<br>GTATTCAATCAGTTACATTTGGTGCTCTGAAATATAAATGACCACCGATG<br>GCTCAGCCCAGCCTGACCTCAAGGGCACGTCTTTACGCCTTGTTTCTCTCT<br>GGTGCAGCTGAGTGTCCCGGAGGGTGTCGAAATGGAGGCTTTTGTAACG<br>AAAGGCGGGTCTGCGAGTGTCCGGATGGGTTCTACGGGCCTCACTGTGA<br>GAAAGCCCTGTGCATACCCCGATGTATGAACGGTGGTCTGTGTGTCACTC<br>CTGGCTTCTGCATCTGCCCCCCTGGATTCTACGGTGTCAACTGTGACAAA<br>GCAAACTGCTCAACCACCTGCTTTAATGGAGGGACCTGCTTTTACCCGGG<br>AAAATGTATTTGCCCTCCTGGACTCGAGGGAGAGCAGTGTGAACTCAGCA<br>AATGCCCCCAACCCTGCCGAAATGGAGGTAAATGCATTGGTAAAAGCAAG<br>TGTAAGTGCCCGAAAGGTTACCAAGGAGACCTGTGCTCTAAGCCCGTCTG<br>CGAGCCTGGCTGTGGTGCCCACGGAACCTGCCACGAACCCAACAAGTGCC<br>AGTGTCGAGAGGGCTGGCACGGCAGACACTGCAATAAGAGGTATGGAGC<br>CAGCCTCATGCATGCCCCGAGGCCAGCAGGCGCCGGGCTGGAGCGACAC<br>ACGCCTTCACTTAAAAAGGCTGAGGATAGAAGGGATCCACCTGAATCCAA<br>TTACATCTGGTGAACCCCTACCCCACCATCTGAAACGGTTCAAGTTACACC<br>GGGTTCACAGCCTTTGTTAACCTTTCGCGTGTTGGATGTTCAAATGCTGT<br>TCATTACACTTTAGAACGCCGGCCTGAATTTTATTAGCTTCATTATAAATC<br>ACTGGGCTGATATCTACTCTTCCTTTTAGGTTTTCTAAGCGTGTCTAGCAT<br>GATGGTATAGATTTTCTTCCTTCAGTCCTTTTGGGACAGATCTTATATTGT<br>GTCAGTTGATCAGGTTAAAAAGAAAAAAAAAATATCTGTCTTTTCAGTGTG<br>TAGTTGACAGATACTTGCAAAATCACAACACATTTGTGGTCTTAGAATGG<br>GGAGTGTTAGAGAGGTTAAACTGGGCAGAGATGCATAAATTACAAGGTT<br>TCGGATAAAGCCAATAGCAGCGTTTAAGCTACAGTATTTCCAATTTTATT<br>GTCAAATATTTGGACATCTGTCTAATTAATACTTCAATTGCCCCCCCCCCA<br>TCTTGAATGCATACAATCTATTTCACCCTTGCTGTTACTCTAGACAGTTCA<br>GTTTTGATGGGGCGGGGGACAAAGTTTAAAAAAATTACACTGAGTTAGC<br>GGCATTTAAACAATATAATATATTGTAAACACGACGAGATAAGGAATATA<br>ATGTATGAAGCCTTTGCATTGGATGGAAGCAATATAATATATTGTAAACA<br>AAACACAGCTCTTACATAGTAAACGTTTTATACTGTTTGTATGTATGAAAT<br>AAAGGTGACGCTTTCAAAAAAAAAAAAAAAAAAAAAAAAAAAAAAAAAA
**iCreb5(bovine Creb5)**	ATGATTTATGAGGAATCCAAGATGAATTTGGAGCAGGAGAGGCCGTTTG<br>TCTGCAGTGCCCCAGGCTGCTCCCAGCGCTTCCCAACAGAGGATCATCTG<br>ATGATTCATAGGCACAAGCATGAAATGACTTTGAAGTTTCCCTCAATAAA<br>AACAGACAATATGTTATCAGATCAAACTCCAACCCCAACGAGATTCCTGA<br>AGAACTGCGAGGAGGTGGGGCTCTTCAGTGAGTTGGACTGCTCCCTAGA<br>GCATGAGTTCAGGAAGGCTCAGGAGGAAGAGAGCAGCAAGCGGAATATC<br>TCGATGCATAACCCCGTTGGTGGGGCCATGGCGGGGCCTGGAGCTCACC<br>AGCTCGGCAGCGCCCGGATGCCCAACCATGACACCAGCGTTGTGATTCAG<br>CAAGCCATGCCGTCACCCCAGTCCAGCTCTGTCATCACGCAGGCCCCTTC<br>CACCAACCGCCAGATCGGGCCTGTCCCAGGCTCTCTATCTTCTCTGCTCCA<br>TCTTCACAACAGACAGAGACAGCCCATGCCAGCCTCCATGCCTGGGACCC

	TGCCCAACCCTACAATGCCGGGCTCTTCTGCCGTCTTGATGCCAATGGAG<br>AGACAAATGTCAGTGAACTCCAACCTCCTGGGAATGCAAGGTCCAAATCT<br>CAGCAACCCCTGTGCTTCTCCCCAAGTCCAGCCAATGCATTCAGAAGCTA<br>AGATGAGGCTGAAGGCTGCATTGACTCACCACCCTGCTGCCATGTCGAAT<br>GGGAATATGAACACCATGGGCCACATGATGGAAATGATGGGCTCCCGGC<br>AGGACCAGACGCCACACCATCACATGCACTCACACCCGCATCAGCACCAG<br>ACACTGCCGGCCCACCACCCCTACCCGCACCAGCACCAGCACCCAGCACA<br>CCACCCTCATCCTCAACCCCATCACCAGCAGAACCACCCACATCATCACTC<br>CCACTCCCACCTTCATGCACACCCAGCACATCACCAGACCTCGCCACACCC<br>ACCCCTGCACTCCGGCACCCAAGCACAGGTTTCACCAGCAACACAACAGA<br>TGCAGCCCACCCAGACAATACAGCCGCCCCAGCCCACAGGGGGGCGCCG<br>GCGAAGGGTGGTGGATGAGGATCCTGACGAGAGGCGGCGGAAATTTCTG<br>GAACGGAACCGGGCGGCCGCCACCCGCTGCAGGCAGAAGAGGAAGGTCT<br>GGGTGATGTCACTGGAAAAGAAAGCAGAAGAGCTCACCCAGACAAACAT<br>GCAGCTTCAGAACGAAGTGTCCATGTTGAAGAACGAGGTGGCACAGCTG<br>AAGCAGCTGTTGTTAACACATAAAGACTGCCCCATAACAGCCATGCAGAA<br>AGAATCACAAGGATATCTCAGTCCAGAGAGTAGCCCTCCTGCAAGTCCCA<br>CCCCCGCATGCTCACAGCAGCAGGTCATCCAGCATAACACCATCACCACT<br>TCCTCGGCAGTCAGCGAGGTGGTAGGAAGCTCTACCCTCAGCCAGCTCAC<br>CACTCACAGAACAGACCTGAACCCCATCCTCGGAGGTTACCCTTACGACG<br>TCCCAGACTACGCTGGCTCCTACCCTTACGACGTCCCAGACTACGCTTACC<br>CTTACGACGTCCCAGACTACGCTTAA
**Runx2**	ATGCGTATTCCTGTAGATCCGAGCACCAGCCGGCGCTTCAGCCCCCCCTC<br>CAGCAGCCTGCAGCCCGGCAAGATGAGCGACGTGAGCCCGGTGGTGGCT<br>GCGCAGCAGCAGCAACAGCAGCAGCAGCAACAGCAGCAGCAACAACAGC<br>AACAGCAACAACAGCAGCAGCAGCAGCAGCAGCAGCAGGAGGCGGCCGC<br>AGCAGCAGCGGCGGCAGCGGCGGCGGCAGCAGCGGCGGCGGCCGCAGT<br>GCCCCGATTGAGGCCGCCGCACGACAACCGCACCATGGTGGAGATCATC<br>GCGGACCACCCGGCCGAACTGGTCCGCACCGACAGTCCCAACTTCCTGTG<br>CTCCGTGCTGCCCTCGCACTGGCGGTGCAACAAGACCCTGCCCGTGGCCT<br>TCAAGGTTGTAGCCCTCGGAGAGGTACCAGATGGGACTGTGGTTACCGT<br>CATGGCCGGGAATGATGAGAACTACTCCGCCGAGCTCCGAAATGCCTCCG<br>CTGTTATGAAAAACCAAGTAGCCAGGTTCAACGATCTGAGATTTGTGGGC<br>CGGAGCGGACGAGGCAAGAGTTTCACCTTGACCATAACAGTCTTCACAAA<br>TCCTCCCCAAGTGGCCACTTACCACAGAGCTATTAAAGTGACAGTGGACG<br>GTCCCCGGGAACCAAGAAGGCACAGACAGAAGCTTGATGACTCTAAACCT<br>AGTTTGTTCTCTGATCGCCTCAGTGATTTAGGGCGCATTCCTCATCCCAG<br>TATGAGAGTAGGTGTCCCGCCTCAGAACCCACGGCCCTCCCTGAACTCTG<br>CACCAAGTCCTTTTAATCCACAAGGACAGAGTCAGATTACAGATCCCAGG<br>CAGGCACAGTCTTCCCCACCGTGGTCCTATGACCAGTCTTACCCCTCCTAT<br>CTGAGCCAGATGACATCCCCATCCATCCACTCCACCACGCCGCTGTCTTCC<br>ACACGGGGCACCGGGCTACCTGCCATCACTGACGTGCCCAGGCGTATTTC<br>AGATGATGACACTGCCACCTCTGACTTCTGCCTCTGGCCTTCCTCTCTCAG<br>TAAGAAGAGCCAGGCAGGTGCTTCAGAACTGGGCCCTTTTTCAGACCCCA<br>GGCAGTTCCCAAGCATTTCATCCCTCACTGAGAGCCGCTTCTCCAACCCA<br>CGAATGCACTACCCAGCCACCTTTACCTACACCCCGCCAGTCACGTCAGG<br>CATGTCCCTCGGCATGTCCGCCACCACTCACTACCACACGTACCTGCCACC<br>ACCCTACCCCGGCTCTTCCCAAAGCCAGAGTGGACCCTTCCAGACCAGCA<br>GCACTCCATATCTCTACTATGGTACTTCGTCAGCATCCTATCAGTTCCCAA<br>TGGTACCCGGGGGAGACCGGTCTCCTTCCAGGATGGTCCCACCATGCACC<br>ACCACCTCGAATGGCAGCACGCTATTAAATCCAAATTTGCCTAACCAGAA

	TGATGGTGTTGACGCTGACGGAAGCCACAGCAGTTCCCCAACTGTTTTGA ATTCTAGCGGCAGAATGGATGAGTCTGTTTGGCGGCCATATTGA
**Wnt5a**	GCTGCTCGCCCGCGCGCGCCGGCGCCCTCTCGGTTCCTGGGCACATTTCC ACGCTATACCAACTCCTCTGCCCGAGTCCGGGCGCCAGTGCTCGCTTCCG CTCCGGGTCGCTGCGCCCACCCGACGCGCCCAGGAGGACTCCGCAGCCCT GCTTTGGATTGTCCCCCAAGGCTTAACCCCGACGCTTCGCTTGAATTCCTC GGCCGCCTTCGCTCGGGTGGCGACTTCCTCTCCGTGCCCCCTCCCCCTCG CCATGAAGAAGCCCATTGGAATATTAAGCCCGGGAGTGGCTTTGGGGAC CGCTGGAGGTGCCATGTCTTCCAAGTTCTTCCTAATGGCTTTGGCCACGT TTTTCTCCTTCGCCCAGGTTGTTATAGAAGCTAATTCTTGGTGGTCTCTAG GTATGAATAACCCTGTTCAGATGTCAGAAGTATATATCATAGGTGCACAG CCTCTCTGCAGCCAACTGGCAGGACTTTCTCAAGGACAGAAGAAACTCTG CCACTTGTATCAGGACCACATGCAGTACATTGGAGAAGGTGCGAAGACA GGCATCAAGGAATGCCAGTACCAGTTCCGGCATCGGAGATGGAACTGCA GCACAGTGGACAATACTTCTGTCTTTGGCAGGGTGATGCAAATAGGCAGC CGAGAGACGGCCTTCACGTACGCGGTGAGCGCAGCTGGGGTGGTGAACG CCATGAGCCGAGCATGCCGGGAGGGCGAGCTGTCTACCTGTGGCTGCAG CCGCGCTGCGCGCCCCAAGGACCTGCCTCGGGACTGGTTGTGGGGCGGC TGCGGAGACAACATCGACTATGGCTACCGCTTCGCCAAGGAGTTCGTGGA CGCTAGAGAAAGGGAACGAATCCACGCTAAGGGTTCCTATGAGAGCGCA CGCATCCTCATGAACTTACACAACAATGAAGCAGGCCGTAGGACAGTATA CAACCTGGCAGATGTAGCCTGTAAGTGTCATGGAGTGTCTGGCTCCTGTA GCCTCAAGACGTGCTGGCTGCAGCTGGCGGACTTCCGGAAGGTGGGCGA TGCCCTCAAGGAGAAGTATGATAGCGCGGCGGCCATGAGGCTCAACAGC CGGGGCAAGCTGGTGCAGGTCAACAGCCGCTTCAACTCCCCGACCACGCA GGACCTGGTCTACATCGACCCCAGTCCGGACTACTGTGTGCGCAACGAGA GCACTGGCTCGCTGGGCACGCAGGGACGCCTGTGCAACAAGACCTCAGA GGGGATGGACGGCTGCGAGCTCATGTGCTGTGGGCGTGGCTATGACCAG TTTAAGACAGTGCAGACCGAACGCTGTCATTGCAAGTTTCACTGGTGCTG CTATGTCAAATGCAAGAAGTGCACGGAGATTGTGGATCAGTTCGTGTGCA AATAGTGGTGTGCCTGCCCTTCACCCAGTCCCACTCCCAGGACCCACTTA TTTATAGAAAGTACAGTGCTTCTGGTTCTTTTTATTTCTCCCCCAAGAATT GCAGCTGGAACCATGTGTTTTGTTTTGTTTTATTTTGTTTTTTCTTTTCTG TTACCATCTAAGAACTCTGTGGTTTATTATTAATATTATAATTAATATTTG GCAATAGTGGGGGAAACTAAGAAAAATATTTATTTTGAGGATCTTTGCAA AGTTAGTACAAAATTTCTTTCTTCTGATGCTACAGGATAAAGGGGAAAAA CTATGTATTCGAACTTAGCTGTGCAGTTGGGGGTTCACATCTAGAAGGTG TAGGAGCCATTTTCTTCTCAAACAGAGAGTCCTTTGAGATGGGTGGTATC CAGGTGAAGGAGGAGGTACAGACCCATGAATAACAGTTCCTGTGACCAA AATGAATTGCAGGTGCTCTGGTACAAAAGATCTTAAATATAGATATATTA AATATACATATATGCCAAAAATACAGAATATGAGACACTCCCTAACCCAG AGGTTACCAGCCTGGTTTTGTGGGTTTTTTGTTTTGTTTTGTTTTTTCTTT TTTTGGGTTTTGTTTGTTTGTTTGTTTGTTTGTATTTTTGGTGTGTGTGTG TGTGTATTTCTAGAATGATCTTTTAGAAGGTACAAGCAAGAATCTCATAT CTTCAGAAGCAGGCATATCATGTATGTTACTGTGTCCCACCTACAGATAC TCCATTCATGAATGGGCCTTTTTCTAACAGTTCATGAATATTGGGGAGCC GGTGGGCTGGGGGAGGGAGGTCCCCAGAAATTAGAAAACTTGAAGTTTC CTACATTGAGGCCATAATCTTGTGTTAGCCCAGCTGATTCTTAATACCAG ACTTTTAGATCCATAAAGGAATTTTTGACTAAAAAAAAAAAAAAAAAA
**Wnt4**	ATGAGCCCCCGTTCGTGCCTGCGGTCCCTGCGACTCCTCGTCTTCGCCGT GTTCTCGGCCGCCGCGAGCAATTGGCTGTACCTGGCCAAGCTGTCATCGG

	TGGGCAGCATCTCCGAAGAGGAGACGTGCGAGAAACTCAAAGGCCTGAT<br>CCAGAGGCAGGTGCAGATGTGCAAACGGAACCTTGAGGTGATGGACTCA<br>GTGCGCCGTGGTGCCCAGCTGGCCATCGAGGAGTGCCAATACCAGTTCC<br>GGAACCGGCGCTGGAACTGTTCCACACTGGACTCCCTCCCTGTCTTTGGG<br>AAGGTGGTGACACAAGGGACCCGGGAGGCGGCCTTTGTATACGCCATCT<br>CTTCAGCAGGTGTGGCCTTTGCAGTGACAAGGGCATGCAGCAGTGGAGA<br>ACTGGAGAAGTGTGGCTGTGACCGGACAGTGCACGGGGTCAGCCCACAG<br>GGCTTCCAGTGGTCAGGATGCTCGGACAACATCGCCTATGGCGTAGCCTT<br>CTCACAGTCCTTTGTGGACGTCCGGGAGAGGAGCAAGGGGGCCTCCTCC<br>AGCCGGGCACTCATGAATCTTCACAACAACGAGGCTGGCAGGAAGGCCA<br>TCTTGACACACATGCGGGTGGAGTGCAAGTGTCACGGG<br>GTGTCGGGCTCCTGCGAGGTAAAGACGTGCTGGCGTGCTGTACCGCCCT<br>TCCGCCAGGTTGGCCACGCGCTAAAGGAGAAGTTTGACGGTGCCACGGA<br>GGTGGAGCCACGACGCGTAGGCTCCTCCCGGGCGCTGGTGCCTCGGAAT<br>GCACAGTTCAAGCCACATACAGATGAGGACCTGGTATACCTGGAGCCTAG<br>CCCGGACTTCTGTGAGCAGGACATCCGCAGTGGCGTGCTAGGCACGAGG<br>GGCCGCACGTGCAACAAGACATCTAAAGCCATTGACGGCTGCGAGCTACT<br>GTGCTGTGGCCGCGGCTTCCACACAGCGCAAGTGGAGCTGGCCGAGCGC<br>TGTGGCTGCAGGTTCCACTGGTGCTGCTTCGTCAAGTGCCGGCAGTGCCA<br>GCGGCTCGTGGAGATGCACACGTGCCGGTGA
**GDF5**	CTAGTGTTTGGTCGTACCAAGAAACGGGACCTGTTCTTTAATGAGATTAA<br>GGCCCGCTCTGGCCAGGATGACAAGACTGTGTATGAATATTTGTTCAGCC<br>AGCGGCGGAAACGCCGGGCCCCATTGGCCAATCGCCAGGGCAAGCGACC<br>CAGCAAGAACCTCAAGGCTCGCTGCAGTCGCAAGGCCTTGCATGTCAACT<br>TCAAGGACATGGGCTGGGACGACTGGATCATCGCACCTCTTGAGTATGA<br>GGCCTTCCACTGCGAAGGACTGTGTGAGTTCCCCTTGCGCTCCCACTTGG<br>AGCCCACAAACCACGCAGTCATTCAGACCCTAATGAACTCTATGGACCCT<br>GAATCCACACCACCCACTTGTTGTGTGCCTACACGGCTGAGTCCTATTAG<br>CATCCTCTTCATCGACTCTGCCAACAACGTGGTGTATAAACAGTACGAGG<br>ACATGGTCGTGGAATCTTGTGGCTGCAGGTAGCAGCACTGGCCCACCTGT<br>CTTCCAGGGTGGCACATCCAGAGACTACCCCCTCCACAGGTTCCTGGAGT<br>AAC

Supplementary Table 6: Genomic sequence confirmation for *Creb5*$^{flox9/+}$ mouse targeting.

Yellow Highlighted: Creb5 locus sequence outside the Left and Right Homologous Arm<br>Blue Highlighted: 270bp Left and Right Homologous Arms<br>Green Highlighted: LoxP Sequence<br>Red Highlighted: Exon 9<br>Gray Highlighted: Sequence in PCR-Blunt vector
ATTGGGCCCTCTAGATGCATGCTCGAGCGGCCGCCAGTGTGATGGATATCTGCAG<br>AATTCAGGCCCATTTGCAGTCTGTAGAGGGAACAGGAAAGAGGACCCCAGGTGCC<br>TTCTGACTGTGCAAGGCTCTTCTGGAGGAGTCCAGGGGTGGCAGTGTCCCACGGG<br>TGTGTTCCACTGAGGTGATTCTTAAACACATTTTGGAAGTAACAGAGCCCACAACC<br>CACGATACAGGTATATATATATATATTATGTGAAAAACAAAACCAAAACAAAACG<br>ATCATGCCAATAACCTTGACCTTACCTGTTTCAACTCGGTAGCCATCCACTGCCAA<br>ATTATGCTCTTTAAAGCATCTTAAATAAAACATTTTTTATTACCTGAAGAAGAAAC<br>AAATGCCCCACCCCCACCCCAACACACACACACACAGCACCCCCAATAACTTCGT<br>ATAGCATACATTATACGAAGTTATGAATTCCTAGGATTCTCCAAATGTCCTGCAGG<br>GAAAATAATCGCCTGCTTGTATCATCAAGCCCAAGTACATGGATTGCAGCTCAAG<br>GTCTCTTGCTCTCACAAGTGTGATTTCATTGCTAATCTCATCCCCTGAGAACAAGT<br>TTCCCTTGCTTACTAAAATGTAACTGGCTTCCTGAGCTTTGCTCTTGTAACCCTGGC<br>TTGGGAGTCTTCCTTACCCAAAATGTCCTTCCCTGTCCCCATCACTTCCTCCCTCTC<br>CTTCCCCTCAGGTTTCACCAGCTACACAACAGATGCAGCCAACCCAGACAATACA<br>ACCGCCCCAGCCCACAGGGGGACGCCGGCGAAGAGTGGTGGATGAGGACCCAGA<br>TGAGAGGCGGAGGAAATTTCTGGAAAGGAACCGGGCCGCCGCCACCCGCTGCAG<br>ACAGAAGAGGAAGGTCTGGGTGATGTCACTGGAAAAGAAAGCTGAAGAGCTCAC<br>CCAGACAAACATGCAGCTTCAGGTGCGGCCCCCATCCCTTCCCCGGCCTCATCAC<br>AGCTGGCGTTTCCTGCCCCTGAGGCTTGTTGACCCAAGCGTCCTACCACACACCTG<br>GCCTCAGAGGAACCCAGGCCCTCTCATCCAGCATCATTAAGTAGTCCGGTACCAT<br>CAGTCCTCCCACTAGAGATGAGGGATGCATGGGACCAAGCACAGAAGCAGGGCTT<br>TGTGCCTTGCTTTGGTGGCTTCTGGCTGTGCTTAAGCAGTCTGGCTCTCTGTTTCCT<br>CATATAACTTCGTATAGCATACATTATACGAAGTTATAAGCTTCTATAATTAGGGA<br>CCAACTTTACAGAGTTGGAGGTGAGATGTGAACCTATGTCCACGGTGCACTTAGC<br>ATGGTCCCTGGCACAGGAGGCTCTTCATCTGGAAGCCATCTAGTGTGGGCACCAG<br>GCATACACCCGATGGTCTGTATTTTTATTTTTAACAGAAAAATATTTTATGTCTTGG<br>CATTGTGTCGGGTTTGTTTGCTCTAAAGAGTAAATGCCTGTTTCCATCTTCATTCCA<br>GCCTCAGCAACATTGGAATCTTGTGTGATTTTTGCTTTCCAGGGGTGGCTTAGCTA<br>GGAACATCCCTGAATTCCAGCACACTGGCGGCCGTTACTAGTGGATCCGAGCTCG<br>GTACCAAGCTTGATGCATAGCTTGAGTATCTAACGCNTCACCTA
