# Supplementary material for: Creb5 coordinates synovial joint formation with the genesis of articular cartilage
Source: Nat Commun. 2022 Nov 26;13:7295. doi: 10.1038/s41467-022-35010-0 (PMC9701237; doi:10.1038/s41467-022-35010-0)
Supplement: Supplementary file 3 — Description of Additional Supplementary Files [file 41467_2022_35010_MOESM3_ESM.pdf]

Description of Additional Supplementary Files:

Supplementary Data 1. Differentially expressed genes in bovine superficial zone articular chondrocytes that had been infected with lentivirus programmed to express either a control shRNA or shCreb5 and cultured in presence of the EGFR ligand, TGFa. The base mean (in 4 biological repeats), log2 fold change in gene expression, p value, and adjusted p value are indicated.

Supplementary Data 2. Differentially expressed genes in bovine superficial zone articular chondrocytes that had been infected with lentivirus programmed to express either a control shRNA or shCreb5 and cultured in the presence of both TGFa and TGFb2. The base mean (in 4 biological repeats), log2 fold change in gene expression, p value, and adjusted p value are indicated.
