# Supplementary material for: Creb5 coordinates synovial joint formation with the genesis of articular cartilage
Source: Nat Commun. 2022 Nov 26;13:7295. doi: 10.1038/s41467-022-35010-0 (PMC9701237; doi:10.1038/s41467-022-35010-0)
Supplement: Supplementary file 6 — Reporting Summary [file 41467_2022_35010_MOESM6_ESM.pdf]

nature portfolio

Corresponding author(s): Andrew Lassar
Cheng-Hai Zhang

Last updated by author(s): Nov 11, 2022

Reporting Summary

Nature Portfolio wishes to improve the reproducibility of the work that we publish. This form provides structure for consistency and transparency in reporting. For further information on Nature Portfolio policies, see our Editorial Policies and the Editorial Policy Checklist.

Statistics

For all statistical analyses, confirm that the following items are present in the figure legend, table legend, main text, or Methods section.

n/a	Confirmed	
☐	☒	The exact sample size (*n*) for each experimental group/condition, given as a discrete number and unit of measurement
☐	☒	A statement on whether measurements were taken from distinct samples or whether the same sample was measured repeatedly
☐	☒	The statistical test(s) used AND whether they are one- or two-sided<br>*Only common tests should be described solely by name; describe more complex techniques in the Methods section.*
☐	☒	A description of all covariates tested
☐	☒	A description of any assumptions or corrections, such as tests of normality and adjustment for multiple comparisons
☐	☒	A full description of the statistical parameters including central tendency (e.g. means) or other basic estimates (e.g. regression coefficient) AND variation (e.g. standard deviation) or associated estimates of uncertainty (e.g. confidence intervals)
☐	☒	For null hypothesis testing, the test statistic (e.g. *F*, *t*, *r*) with confidence intervals, effect sizes, degrees of freedom and *P* value noted<br>*Give P values as exact values whenever suitable.*
☒	☐	For Bayesian analysis, information on the choice of priors and Markov chain Monte Carlo settings
☒	☐	For hierarchical and complex designs, identification of the appropriate level for tests and full reporting of outcomes
☒	☐	Estimates of effect sizes (e.g. Cohen's *d*, Pearson's *r*), indicating how they were calculated

Our web collection on statistics for biologists contains articles on many of the points above.

Software and code

Policy information about availability of computer code

Data collection	The RNA-Seq data were generated on Illumina NextSeq 500 System.
Data analysis	The sequencing data were processed using the bcbio-nextgen pipeline (v 1.2.4) with bovine reference genome UMD 3.1. The basic quality of the data was checked using FastQC (v 0.11.8) and mapping metrics were assessed by aligning the reads to reference genome using STAR (v 2.6.1d). Transcript expression was quantified using Salmon (v 0.14.2) and aggregated for gene-level counts using tximport (v 1.14.2). Differentially expressed (DE) genes between treatment pairs were identified using DESeq2 (v 1.24.0) with FDR threshold of 0.05 in R (v 3.6.3). The volcano plots were generated using ggplot2 (v 3.3.2). GraphPad Prism 9 was employed for statistical analyses using two-tailed Student's t-test.

For manuscripts utilizing custom algorithms or software that are central to the research but not yet described in published literature, software must be made available to editors and reviewers. We strongly encourage code deposition in a community repository (e.g. GitHub). See the Nature Portfolio guidelines for submitting code & software for further information.

Data

Policy information about availability of data

All manuscripts must include a data availability statement. This statement should provide the following information, where applicable:

- Accession codes, unique identifiers, or web links for publicly available datasets
- A description of any restrictions on data availability
- For clinical datasets or third party data, please ensure that the statement adheres to our policy

Data Availability
All primary RNA-Seq data sets generated and analyzed during the current study are available in the GEO repository (accession GSE181883):
Go to https://www.ncbi.nlm.nih.gov/geo/query/acc.cgi?acc=GSE181883

Human research participants

Policy information about studies involving human research participants and Sex and Gender in Research.

Reporting on sex and gender	*Use the terms sex (biological attribute) and gender (shaped by social and cultural circumstances) carefully in order to avoid confusing both terms. Indicate if findings apply to only one sex or gender; describe whether sex and gender were considered in study design whether sex and/or gender was determined based on self-reporting or assigned and methods used. Provide in the source data disaggregated sex and gender data where this information has been collected, and consent has been obtained for sharing of individual-level data; provide overall numbers in this Reporting Summary. Please state if this information has not been collected. Report sex- and gender-based analyses where performed, justify reasons for lack of sex- and gender-based analysis.*
Population characteristics	*Describe the covariate-relevant population characteristics of the human research participants (e.g. age, genotypic information, past and current diagnosis and treatment categories). If you filled out the behavioural & social sciences study design questions and have nothing to add here, write "See above."*
Recruitment	*Describe how participants were recruited. Outline any potential self-selection bias or other biases that may be present and how these are likely to impact results.*
Ethics oversight	*Identify the organization(s) that approved the study protocol.*

Note that full information on the approval of the study protocol must also be provided in the manuscript.

Field-specific reporting

Please select the one below that is the best fit for your research. If you are not sure, read the appropriate sections before making your selection.

[x] Life sciences [ ] Behavioural & social sciences [ ] Ecological, evolutionary & environmental sciences

For a reference copy of the document with all sections, see nature.com/documents/nr-reporting-summary-flat.pdf

Life sciences study design

All studies must disclose on these points even when the disclosure is negative.

Sample size	The number of independent biological repeats for each experimental observation are indicated in the figure legends.
Data exclusions	No data was excluded.
Replication	The number of independent biological repeats for each experimental observation are indicated in the figure legends.
Randomization	Not relevant to this study.
Blinding	To quantitate the number of flat cells in the superficial zone of the articular cartilage, we counted (in a double-blinded fashion) the number of flat cells present in the superficial most top two layers of cells, normalized to the length of the measured articular cartilage surface.

Reporting for specific materials, systems and methods

We require information from authors about some types of materials, experimental systems and methods used in many studies. Here, indicate whether each material, system or method listed is relevant to your study. If you are not sure if a list item applies to your research, read the appropriate section before selecting a response.

Materials & experimental systems

n/a	Involved in the study
☐	☒ Antibodies
☐	☒ Eukaryotic cell lines
☒	☐ Palaeontology and archaeology
☐	☒ Animals and other organisms
☒	☐ Clinical data
☒	☐ Dual use research of concern

Methods

n/a	Involved in the study
☒	☐ ChIP-seq
☒	☐ Flow cytometry
☒	☐ MRI-based neuroimaging

Antibodies

Antibodies used	Antibodies employed in this study are listed in Supplementary Tables 2 and 3.
Validation	Catalogue numbers and vendors of all antibodies employed are listed in Supplementary Tables 2 and 3.

Eukaryotic cell lines

Policy information about cell lines and Sex and Gender in Research

Cell line source(s)	HEK 293T cells were employed to generate lentivirus. 293T/17 [HEK 293T/17] (ATCC® CRL-11268™) were obtained from ATCC.
Authentication	none
Mycoplasma contamination	Cell lines were not tested for mycoplasma contamination.
Commonly misidentified lines (See ICLAC register)	not relevant

Animals and other research organisms

Policy information about studies involving animals; ARRIVE guidelines recommended for reporting animal research, and Sex and Gender in Research

Laboratory animals	Mice were housed in a state of the art, full barrier animal facility. Animal experiments in this study were conducted in accordance with the guidelines of the Harvard Medical Area (HMA) Standing Committee on Animals (i.e., Harvard Medical School office of the IACUC).
Wild animals	Not relevant.
Reporting on sex	Studies were performed with both sexes of mice. We have reported the sex of animals analyzed that were sacrificed at P14 or older.
Field-collected samples	Not relevant.
Ethics oversight	All work with vertebrate animals was approved by the Harvard Medical School Institutional Animal Care and Use Committee (IACUC).

Note that full information on the approval of the study protocol must also be provided in the manuscript.
